# Leaf trait variation across Mediterranean forest endemics: drivers and evidence for lower resource acquisition ability than in widespread forest congeners

**DOI:** 10.3389/fpls.2025.1664759

**Published:** 2025-09-19

**Authors:** Federico Selvi, Elisa Carrari, Giandiego Campetella, Roberto Canullo, Stefano Chelli, Andrea Coppi, Emmanuele Farris, Alfredo Maccioni, Francesco Mascia, Nicola Postiglione, Leonardo Rosati, Ilaria Santi, Camilla Wellstein, Marco Cabrucci, Cristina Gasperini

**Affiliations:** ^1^ Department of Agriculture, Food, Environment and Forestry, University of Florence, Florence, Italy; ^2^ School of Biosciences and Veterinary Medicine, University of Camerino, Camerino, Italy; ^3^ Department of Biology, University of Florence, Florence, Italy; ^4^ Department of Chemical, Physical, Mathematical and Natural Sciences, University of Sassari, Sassari, Italy; ^5^ Ecosystem of Innovation for Next Generation Sardinia (e.INS), Sassari, Italy; ^6^ Department of Health Sciences, University of Basilicata, Potenza, Italy; ^7^ Faculty of Agricultural, Environmental and Food Sciences, Free University of Bozen-Bolzano, Bolzano, Italy

**Keywords:** endemic plants, forest habitats, functional divergence, leaf traits, Mediterranean flora, resource-use strategies, understorey species

## Abstract

**Introduction:**

Despite their biogeographical relevance, the trait space exploited by endemic plants of Mediterranean forests remains largely unknown. Understanding their functional divergence from widespread congeners is key to explaining their restricted distribution, ecology, and resource-use strategies.

**Methods:**

Here, we analyzed interspecific variability in leaf economic traits capturing plant strategies of resource-use such as leaf area (LA), specific leaf area (SLA), leaf mass per area (LMA), leaf dry matter content (LDMC), leaf nitrogen content per unit dry mass (Nmass) and carbon to nitrogen ratio (C:N ratio), across 45 endemic taxa of Mediterranean forests. The influence of environmental variables and the phylogenetic signal of traits were examined to identify the main drivers. Next, we performed paired comparisons in 27 endemic-non endemic pairs, with allopatric, parapatric and sympatric distribution.

**Results and discussion:**

Overall, trait variability within endemics was remarkably ample, reflecting their diversity in functional types, phylogenetic relationships and biogeographical contexts. Endemics were widely distributed along the resource use gradient associated with LA, LMA and Nmass. Herbaceous taxa showed more resource-acquisitive trait values and prevalence of C and R strategies, while woody endemics were more resource-conservative and stress-tolerant. Traits showed a phylogenetic signal of variable intensity depending on the metrics, with Pagel’s λ approaching the Brownian model for LA and LMA. Environmental factors variously influenced trait variation. LA decreased with temperature and depended on forest type, while LDMC decreased with latitude and precipitation. LMA increased with temperature and varied with ecoregion and forest type, while Nmass decreased with latitude and increased with precipitation. Species pairs analysis revealed a negative effect of the endemic condition on LA, but positive on LMA. Compared with widespread congeners, this pointed to a lower acquisitive ability and stronger resource conservation attitude, also confirmed by CSR strategies. Differences in LA and LMA within allopatric and parapatric pairs were larger than in sympatric pairs, suggesting the role of vicariance in key leaf trait divergence. In advancing our understanding of the functional and ecological characteristics of Mediterranean endemic forest plants, this study may help to predict the effects of the increasing pressures to their habitat and support strategies for their conservation.

## 1 Introduction

The reasons why endemic plant species have a more or less restricted range with respect to widespread congeners are a central issue in ecology and biogeography (Lavergne et al., 2004). While many studies have focused on the relationships and conservation of endemic plants, only a few investigations have delved into the factors determining their inherently limited ability to spread over wider territories (Hobohm, 2014). It is well established that a multiplicity of historical, ecological and biological circumstances, often variously combined together, can result in a limited dispersal capacity and a restricted distribution range of a given species (Song and Li, 2023). Understanding these factors is increasingly important to help predicting the effects of global changes on endemic plants worldwide, and support strategies for their conservation (Damschen et al., 2012; Erfanian et al., 2021; Erlandson et al., 2021; Hobohm, 2014). In the Mediterranean biodiversity hotspot, narrow endemics are a significant proportion of the rich native flora (Quézel and Médail, 2003). It was highlighted that these taxa are most often ecologically divergent with respect to widespread congeners, being restricted to harsh habitats such as steep rocky slopes with open vegetation and low species richness (Thompson, 2020; Thompson et al., 2005). Niche specialization and adaptation in response to stressful conditions and selective pressures have been generally assumed to be major drivers for their origin and persistence, allowing them to colonize more extreme habitats and to escape from competition and anthropogenic disturbance (Coppi et al., 2014; Hand et al., 2017; Médail and Verlaque, 1997; Totté et al., 2015).

Niche specialization and ecological divergence are less likely to occur under the more favorable conditions of forest habitats. This explains, at least in part, the minor proportion of narrow-ranged endemics in temperate European forests (Quézel and Médail, 2003; Rackam and Grove, 2001). Stronger interspecific competition and, often, more intense disturbance in woodlands are further factors contributing to the low proportion of forest endemics in Europe (Bruchmann, 2011; Hobohm, 2014). In contrast, the Mediterranean flora includes substantial endemic woody and herbaceous plants within forest habitats, found in closed interiors, open canopies, gaps, and edges (Selvi et al., 2023). Despite the conservation relevance, rarity, and isolated phylogenetic position of many of these taxa, their biology and ecology remain largely unknown. Recently, Selvi et al. (2023) provided baseline statistics for over 130 taxa with narrow range across Italian forests, mainly herbaceous but also woody, either “paleo-” or “neo-” endemics, highlighting the current lack of information about their functional characteristics and ecological strategies.

The functional trait approach for the investigation of Mediterranean endemic taxa has been poorly adopted so far, although it can provide insights into the factors involved in their restricted range in terms of plant fitness and resource use strategies. Evidence from mainly non-forest taxa indicates that these plants have a smaller stature than widespread congeners, yet they do not significantly diverge in specific leaf area, leaf nitrogen content, leaf dry matter, or maximum photosynthetic rate (Hand et al., 2017; Lavergne et al., 2004). Overall, this pointed to a lower competitive ability, based on smaller stature, but similar attitudes in the acquisition and use of resources in the taxa analyzed in the above studies. However, whether these findings can be generalized to endemics specialized for a different macro-habitat, such as temperate forests, remains still unclear. Hence, little inference can be made on the factors behind their restricted distribution in terms of competitive ability for light, nutrients and water.

Leaf traits play a critical role in plant growth and survival, largely determining the functional space explored by plants in terms of resource use (Garnier et al., 2015; Niinemets, 2020; Niklas et al., 2023; Wright et al., 2004). Analyzing these traits in forest endemic species and unveiling the range of interspecific variability between them can provide insights into their ecological strategies and position within the leaf economic spectrum (Wright et al., 2004). Moreover, investigating the dependency of traits on environmental and main habitat factors (latitude, altitude, climatic variables, forest type, dominant tree species and others) on the one hand, and on phylogenetic constraints on the other, is key to infer the influence of the evolutionary history of the taxa and the current conditions of their environment on their variation (Garnier et al., 2015).

Several forest endemics in the Mediterranean have widespread (non-endemic) related taxa inhabiting similar habitats across larger continental areas. This circumstance offers the opportunity to compare trait divergence in endemic and non-endemic species pairs, excluding the effect of ecological specialization and under similar constraints of competitive interactions with other species of the canopy and understory communities. The question of whether endemics and widespread congeners differ in traits, resource use strategies and competitive ability can thus be addressed specifically for forest species, unlike in previous studies (Hand et al., 2017; Lavergne et al., 2004; Thompson, 2004; Thompson et al., 2005; Totté et al., 2015). If present, functional divergence and differences in resource use within these species pairs cannot be directly related to clear differences in the light regimes, since both taxa, endemic and non-endemic, share a similar adaptation to shade, a major driver of leaf trait variability (Liu et al., 2016; Poorter et al., 2019; Valladares et al., 2016). Instead, trait divergence may result from evolutionary and biogeographic processes within each species pair, potentially resulting in different resource use attitudes. For this purpose, accounting for the distribution pattern of species in multiple pairs, e.g. whether allopatric, parapatric or sympatric, can help to unravel the role of geographic separation and vicariance in trait variation.

To bring light on the above issues, we measured six core leaf traits capturing plant strategies related to resource use (leaf area, specific leaf area, leaf mass per area, leaf dry matter content, nitrogen mass and C:N ratio) in 45 endemic forest taxa of vascular plants in distant angiosperm and conifer clades, either woody or herbaceous, most of which have not been examined for functional traits so far. These taxa are restricted to parts of the Italian peninsula, especially the southern regions and the islands of Sicily and Sardinia, which are well known centers of plant endemism of high biogeographical relevance, significantly contributing to the diversity of the Mediterranean flora (Médail and Quézel, 1999). Remarkable examples of narrow-ranged and phylogenetically distinctive forest endemics in these areas are the umbellifers *Petagnaea gussonei*, *Cryptotaenia thomasii* and *Heptaptera angustifolia*, the composite *Rhaponticoides centaurium*, the borage *Aegonychon calabrum* or the shrub *Rhamnus persicifolia*. This allowed us to open a window on the range of interspecific variation in leaf traits across Mediterranean endemic forest plants, to infer their position in the Leaf Economic Spectrum and to get insights into their resource use strategies and competitive abilities. Moreover, exploring the influence of a set of unrelated environmental and geographical variables for each sampled species/population and the phylogenetic signal of each trait made it possible to evaluate the role of either ecological or evolutionary constraints on trait variation across the taxa. In addition, expanding trait analysis to twenty widespread and closely related taxa of the same functional type and from similar forest habitat allowed phylogenetically independent comparisons to unveil the levels and patterns of variability in 27 species pairs, also in relation to the model of distribution of the two species in each pair, e.g. whether allopatric, sympatric or parapatric. Based on previous studies, we could test the hypothesis that endemics show no or little divergence from congeners in traits related to resource use such as leaf area, leaf mass per area and nitrogen content, as well as in terms of CSR strategies. Finally, we could explore whether trait divergence is overall lower in sympatric species pairs (with overlapping ranges) than in allopatric or parapatric pairs (with separate ranges), due to geographic and/or ecological vicariance in distinct ecoregions. Overall, this study contributes to a better knowledge of Mediterranean endemic plants and to the recent perspective of integrating the functional approach to the investigation of biodiversity, considering the influences of environment and evolution under environmental change (Liu et al., 2024).

## 2 Materials and methods

### 2.1 Species selection

We built a dataset including 45 endemic taxa (37 species and 8 subspecies) in a wide range of plant families (27) and genera (37) of vascular plants, including conifers, eu-dicot and monocot angiosperms. The dataset includes woody (trees and shrubs; Raunkiaer’s life-forms P scap, P caesp, NP, n=10) and herbaceous taxa (perennial herbs: geophytes and hemicryptophytes, annual herbs: therophytes; n=35), sampled across seven Italian regions, including Sicily and Sardinia (**Supplementary Figure 1**). Twenty taxa are illustrated in **Supplementary Figure 2**. Taxon selection was based on Selvi et al. (2023) who provided a full list and dataset of Italian forest endemics, e.g., taxa that are restricted to Italy or Italy and Corsica (France), in line with the recent checklist of the native Italian flora (Bartolucci et al., 2024) and the portal to the Flora of Italy (https://dryades.units.it/floritaly/). The selected species represent a relatively broad range of phylogenetic lineages and are all typical of forest habitats (guilds 1.1 and 1.2 of Heinken et al. (2022) and Selvi et al. (2023)). Also, all endemics analyzed here are found in forest habitats listed in the EUNIS classification of Terrestrial Habitats (EEA, 2021, as “forest and other wooded land”, T code). Distribution ranges of the selected taxa span from very local within a single Italian region (e.g. *Vicia brulloi, Rhamnus persicifolia*) to relatively wide across several Italian peninsular regions (e.g. *Melampyrum italicum*, *Digitalis micrantha*).

To compare endemics vs sister widespread taxa and allow independent evaluations of trait variation in relation to range size we included in the dataset 20 widespread (non-endemic) woody and herbaceous species in 18 families and 19 genera, resulting in a total of 65 taxa and 27 pairs of taxa analyzed. “Widespread” taxa were distributed across large parts of continental Europe and beyond (see “chorotype” in **Supplementary Table 1**), with a range size many-fold larger than that of the related endemic; these taxa were selected based on the following criteria: i) native presence in Italy, ii) systematic and phylogenetic relatedness as resulting from standard floras (especially Pignatti et al. (2017), monographic works and phylogenetic analyses of given taxonomic groups when available, iii) same Raunkiaer’s life-form and functional type, and iv) clear preference for forest habitat (guilds 1.1 or 1.2 of Heinken et al. (2022) or based on personal experience). In this way, we largely excluded habitat and growth form as factors affecting trait variation. To account for the distribution relationship of the two taxa in each pair, we classified the 27 pairs in three groups: i) allopatric (ranges of the two taxa not overlapping and sharply disjunct, e.g. *Rhamnus persicifolia* vs. *R. cathartica*; n=10), ii) sympatric (range of the endemic totally or largely overlapping with that of the widespread congener, e.g. *Aegonychon calabrum* vs. *A. purpureocaeruleum*, n=9), iii) parapatric (ranges not overlapping but in contact or in close proximity at their limits, e.g. *Cardamine battagliae* vs. *C. heptaphylla*, n=8).

The full list of the 65 taxa included in this study is given in **Supplementary Table 1**.

### 2.2 Field sampling of plant material

Sampling campaigns were conducted in the years 2023 and 2024, in spring and early summer to collect fully developed leaves. We analyzed one population per taxon (both endemics and non-endemics), as this study focused on interspecific variation. The selected population was randomly chosen from those in representative forest sites and habitats for the species, without signs of recent disturbance (e.g. fire, clear-cuttings, windthrow areas). When more populations were present, the most easily accessible one was selected for sampling. Twenty leaves were collected from each site/population, following standard protocols (Kleyer et al., 2008; Pérez-Harguindeguy et al., 2013). At each sampling site, we collected two healthy leaves from ten randomly selected individuals at least 10 m from each other, usually one leaf from the base and one from the upper part of the fertile stem. Two basal leaves were collected in species without well distinct cauline leaves (e.g. *Crocus*). For trees and shrubs, leaves were collected from one-year old twigs in the outer parts of the crown that were best exposed to light. In the case of a few species (e.g. *Paeonia mascula*), less than 20 leaves were collected due to the low number of individuals in the population. In total, 1269 leaves (878 “endemic”, 391 “non-endemic”) were collected and included in the dataset. One specimen for each sampled population was gathered to serve as a voucher in the *Herbarium Centrale Italicum* at the Museum of Natural History of the Florence University (FI-H.C.I). Geographical details of the sampling sites for each taxon are given in **Supplementary Table 1**.

### 2.3 Leaf trait selection and measurement

We analyzed the following traits: i) Leaf area (LA, mm^2^), which is strongly related to photosynthesis rate, water and heat balance and leaf temperature (Cornelissen et al., 2003; Díaz et al., 2016; Falster and Westoby, 2003); ii) Specific Leaf Area (SLA), and its inverse, Leaf Mass per Area (LMA, g.m^-2^), quantifying the combined thickness and density of a leaf and representing the plant investment per area of light intercepting surface (Díaz et al., 2016; Evans and Poorter, 2001; Niinemets, 2001; Wright et al., 2004); SLA and LMA are powerful indicators of a species resource-use strategy and its ability to balance light capture with water conservation (Poorter et al., 2009; Gasperini et al., 2025); iii) Leaf Dry Matter Content (LDMC, g.g^-1;^ after Díaz et al., 2022), a proxy for leaf density, negatively associated with relative growth rate and photosynthetic rate, but positively with nutrient retention (Cornelissen et al., 2003; Poorter and Garnier, 2007); iv) Leaf Nitrogen content per unit Mass (Nmass, mg.g^-1^), a good indicator of plant photosynthetic capacity, respiration and growth rate (Wright et al., 2004), and v) C:N ratio (C:N), negatively correlated with relative growth rate, photosynthetic rates, reflecting higher concentration of Rubisco and other photosynthetic proteins in the leaf (Field and Mooney, 1986). Leaf area, fresh and dry weight (including the petiole) were determined as in the LEDA protocol (Kleyer et al., 2008). Fresh weight was determined as soon as leaves were brought to the laboratory, using a precision balance (Toledo Mettler with sensitivity=0.1mg); next, leaves were scanned (1653 × 2338 pixels, 200 dpi), to allow area measurement with Lafore (Lehsten, 2005). Leaves were then stored and allowed to dry for at least two weeks at room temperature. Samples were further dried in the oven for 72 hrs at 40°C to determine dry weight. For each taxon, one basal and one cauline leaf were collected from five randomly chosen individuals and pooled together for C and N content analysis. These were performed through high temperature combustion at 1000°C using an elemental analyzer (EA 1110 CHNS-O-CE).

Plant vegetative height (H, cm) was measured on herbarium specimens for the herbaceous taxa and visually estimated on two individuals in the field for tall shrub and tree species; all plant heights were checked using *Flora d’Italia*, 2nd edition (Pignatti et al., 2017). At present, availability of trait data in national and global databases is very low for the Italian endemic taxa (no data for 26 taxa out of 45), while most of the widespread congeners are covered for at least one trait (based on TRY (Kattge et al., 2020), February 2025; Chelli et al., 2025).

### 2.4 Environmental variables

Trait variation was analyzed in relation to biogeographical and environmental variables potentially exerting a selective pressure on leaf structure, including: i) latitude; ii) elevation above sea level; iii) the 19 WorldClim bioclimatic variables related to temperature and precipitation (BIO1–BIO19); these variables describe annual averages, measures of seasonality, the warmest and coldest temperatures, the driest and wettest months and the overall variability in temperature and precipitation. These data were extracted from the WorldClim database (Fick and Hijmans, 2017) for all species using the ‘getData’ command from the ‘raster’ package (Hijmans, 2015); iv) main soil type, using the geological WMS map of the national geoportal of the Ministry of the Environment and Energy Security; v) ecoregion according to Blasi et al. (2014), to account for the different biogeographical contexts of the analyzed endemics; vi) tree shade casting ability (SCA) of the dominant tree species in the forest sites where understorey species were sampled, as a proxy for the light regime in their habitat (Leuschner and Ellenberg, 2017; Depauw et al., 2021). SCA values range from 1 (lowest shading ability) to 5 (highest shading ability) and were mostly retrieved from Käber et al. (2021); the tree species not included in previous assessments (e.g. *Quercus ilex*) were scored based on personal experience. For understory populations sampled at the forest edge or in gaps with reduced canopy cover, the SCA value of the dominant tree projecting its shade on the population was multiplied by 0.5; vii) major forest type according to the following classification: evergreen, deciduous broadleaf, hygrophilous, beech, coniferous, open forest. Environmental variables for each taxon are given in **Supplementary Table 2**.

### 2.5 Data analysis

#### 2.5.1 Leaf traits

To assess the significance of differences between woody and herbaceous endemic taxa, we first calculated the mean, variance, coefficient of variation, skewness, and kurtosis for each trait. We then used Mann-Whitney U tests to compare each trait between these two groups. The Pearson’s product moment correlation coefficient was applied to test the correlation between the traits. Next, Principal Component and multifactorial Analyses using LA, LDMC, LMA, Nmass and C:N were applied to summarize and display the position of the 45 endemics in relation to the main axes of leaf functional variation. Principal component analysis (PCA) was performed for the 45 species using the ‘prcomp’ function in package ‘stats’ and visualized using the ‘FactoMineR’ and ‘factoextra’ packages. All response variables were scaled to unit variance before the analysis. The normal distribution of all variables was tested with the ‘shapiro.test’ function and log-transformed when needed. Next, the application Phenospace (Segrestin et al., 2020) was used to visualize the distribution of the endemics and the 27 related congeners in the trait space determined by variation across the 2214 species analyzed in Díaz et al. (2016). To this purpose, we used the traits representing the leaf economics spectrum, LA, LMA, and Nmass, and H (Height) as a measure of the size of the whole plant.

The relative position of the endemics and of the widespread congeners in the CSR plant strategy scheme (Grime, 2001) was assessed using StrateFy (Pierce et al., 2017). This tool allows the calculation of the CSR components of single leaf samples starting from leaf area, fresh weight and dry weight (Santi et al., 2025).

#### 2.5.2 Environmental variables

The effects of the environmental and bioclimatic variables on leaf traits (LA, LMA, LDMC, Nmass, and C:N) were investigated using a linear model approach (command lm in package stats, R Core Team, 2023). A two-step selection was done to reduce the number of potential explanatory variables. First, PCA using the prcomp function from the ‘factoextra’ package (Kassambara and Mundt, 2020) was applied to identify the bioclimatic variables more strongly associated with the first two principal components. Mean annual air temperature (MAT or BIO1) and mean annual precipitation (MAP or BIO12), were most correlated with the first component (r = -0.29 and + 0.28, respectively), which explained 40.5% of the total variance (vs 16% of the 2nd component). These two variables are given for each endemic population in **Supplementary Table 2**. Next, MAT and MAP were included, along with all other environmental variables transformed into numbers, in a correlation analysis using the cor function from the corrplot package (Wei and Simko, 2024). This step was performed to identify multicollinearity among variables and allowed to exclude longitude and elevation, which exhibited correlations > |0.5| with MAT. As a result, five response variables were analyzed using the following linear model:


Y∼Latitude+ecoregion+forest type+soil type+SCA:W/H+MAT+MAP.

The variable SCA was included in interaction with the factor W/H (Woody/Herbaceous) describing the main plant functional group, e.g. woody or herbaceous, as SCA values were not assigned to six woody endemic species that formed the tree canopy cover at the sampling sites (*Abies nebrodensis*, *Acer cappadocicum* subsp. *lobelii*, *Alnus cordata*, *Pinus nigra* subsp. *laricio*, *Rhamnus persicifolia*, *Salix arrigonii*). Other interactions were not included for the sake of simplicity. The most parsimonious model (single best model) for each response variable was selected based on the Akaike Information Criterion (AIC) with the dredge-function of the package MuMIn (Bartoń, 2019). All continuous explanatory variables were scaled to facilitate comparisons. For the categorical variables included in the selected models, group differences were assessed using one-way ANOVA followed by pairwise comparisons with emmeans (package emmeans); when assumptions of normality and homoscedasticity were not satisfied we applied the Kruskal–Wallis test (kruskal.test, package stats) followed by Dunn’s *post hoc* test (dunnTest, package FSA).

#### 2.5.3 Species pairs analysis

Species pairs were analyzed with linear mixed effects models (LMMs) with a Gaussian distribution and using the following equation: Variable ~ IT_endemic + (1 | pair).

The models were fitted using the package ‘lme4’ (Bates et al., 2015) separately for woody and herbs with endemic vs non-endemic as fixed factors (IT_endemic: two categorical variables) and pair as random effect term (27 pair categories). In all models, residuals were evaluated for normality and homogeneity by a visual check of the model assumptions (normality of residuals, normality of random effects, homogeneity of variance). For all models, we computed the proportion of variance explained by the fixed effects of the model (marginal R^2^) and by both random and fixed effects (conditional R^2^; Nakagawa and Schielzeth, 2013).

The mean endemic vs non-endemic difference in LA, LMA, LDMC and Nmass within each of the three distribution groups (allopatric, parapatric, sympatric) was determined as the mean of the differences in each pair for each group, for each trait. The significance of the differences between the three groups was then tested using ANOVA and *post-hoc* Tukey test.

#### 2.5.4 Phylogenetic signal

A well-resolved phylogenetic tree of the 65 taxa in our dataset was obtained using V.PhyloMaker, the function *ape* for scenario selection (1) and *ggtree* for tree plotting (**Supplementary Figure 3**). The phylogenetic signal of LA, LMA, LDMC and Nmass (e.g. the tendency of closely related species to resemble each other more than expected by chance; Blomberg and Garland (2002)) was determined by testing the null hypothesis that these traits evolved independently of phylogenetic relationships between the 45 endemics. Three statistical metrics were used for this purpose: i) Pagel’s λ (Pagel, 1999), ii) Blomberg’s K (Blomberg et al., 2003), and iii) Abouheif’s Cmean (Abouheif, 1999); all were calculated using the R package phylosignal (Keck et al., 2016). Concerning the first metric, λ = 0 indicates no signal while λ = 1 points to phylogenetic signal under a Brownian model of trait evolution. Similarly, Blomberg’ K indicates no signal when = 0, while K > 1 indicates intense signal, with closely related species resembling each other more than expected under the Brownian model; 0 < K < 1 indicates that closely related species resemble each other less than expected, and K = 1 indicates phylogenetic signal as expected by the Brownian model. Although the use of Blomberg’s K has recently increased, it was recently demonstrated that Pagel’s λ is a more appropriate measure to test phylogenetic signal in several circumstances (Avila-Lovera et al., 2023). The third metric, Cmean, is not model-based but represents an autocorrelation measure of the covariation of trait values across species in relation to the phylogenetic distance between the species; Cmean = 1 indicates strong similarity across close relatives and Cmean = − 1 indicates strong negative associations between species and trait similarity. Phylogenetic correlograms were built to visualize how the above traits are autocorrelated at different lags of phylogenetic distance among the taxa. Finally, the Local Moran’s Index (LMI) was computed to detect autocorrelation hotspots across the endemics phylogeny. LMI can show significant positive values when the trait is more similar among closely relatives than expected by chance, or negative values when the trait is more divergent among distantly relatives than expected by chance (Keck et al., 2016). The lipaMoran function was used to determine LMI and autocorrelation values were plotted with the dotplot.phylo4d function in the Phylosignal package. All analyses were performed in R version 4.0.3 (R Core Team, 2023).

## 3 Results

### 3.1 Leaf trait variation in the forest endemics

Trait values across the endemics showed a broad variability, high kurtosis and skewness especially for LA and LMA; Nmass and C:N were comparatively less variable (**Table 1**). Leaf area was significantly higher in herbaceous than in woody taxa (*p* < 0.001; **Supplementary Table 3**), reaching a minimum in the conifer *Abies nebrodensis* and a maximum in the herb *Rhaponticoides centaurium* (see **Supplementary Table 3** for species means and standard deviations for each trait). Leaf mass was instead significantly higher in the woody taxa (*p* < 0.001), especially in the two conifers. An approximately ten-fold variation occurred in this trait among herbs, with low values in *Asyneuma Petagnaea* and *Cryptotaenia* and relatively high in *Echinops*; in *Helleborus*, a species with long and thick leaf petiole, variation was about 30-fold. Nitrogen content (Nmass) was slightly higher in herbaceous than in woody taxa (p < 0.05), contributing to a significantly lower C:N ratio in the former group (*p* < 0.001). The C:N ratio was in fact inversely related with Nmass (Pearson r = -0.78) and positively with LDMC (r = 0.52), while no covariation was found between the other traits (**Supplementary Figure 4**).

**Table 1:** Main statistical moments for each leaf trait across the 45 endemic taxa analyzed.

Statistics	LA mm^2^	LDMC g.g^-1^	LMA g.m^-2^	Nmass mg.g^-1^	C:N
Min	34.7	0.058	14.6	7.39	8.66
Max	34197	0.594	464.9	50.32	64.29
Mean	4691	0.231	64.1	26.83	18.60
Variance	52546610	0.0128	6291.2	92.05	101.01
Skewness	2.7604	1.4243	3.65	0.36	2.84
Kurtosis	10.59	4.9305	17.05	2.88	12.47

**Figure 1:**
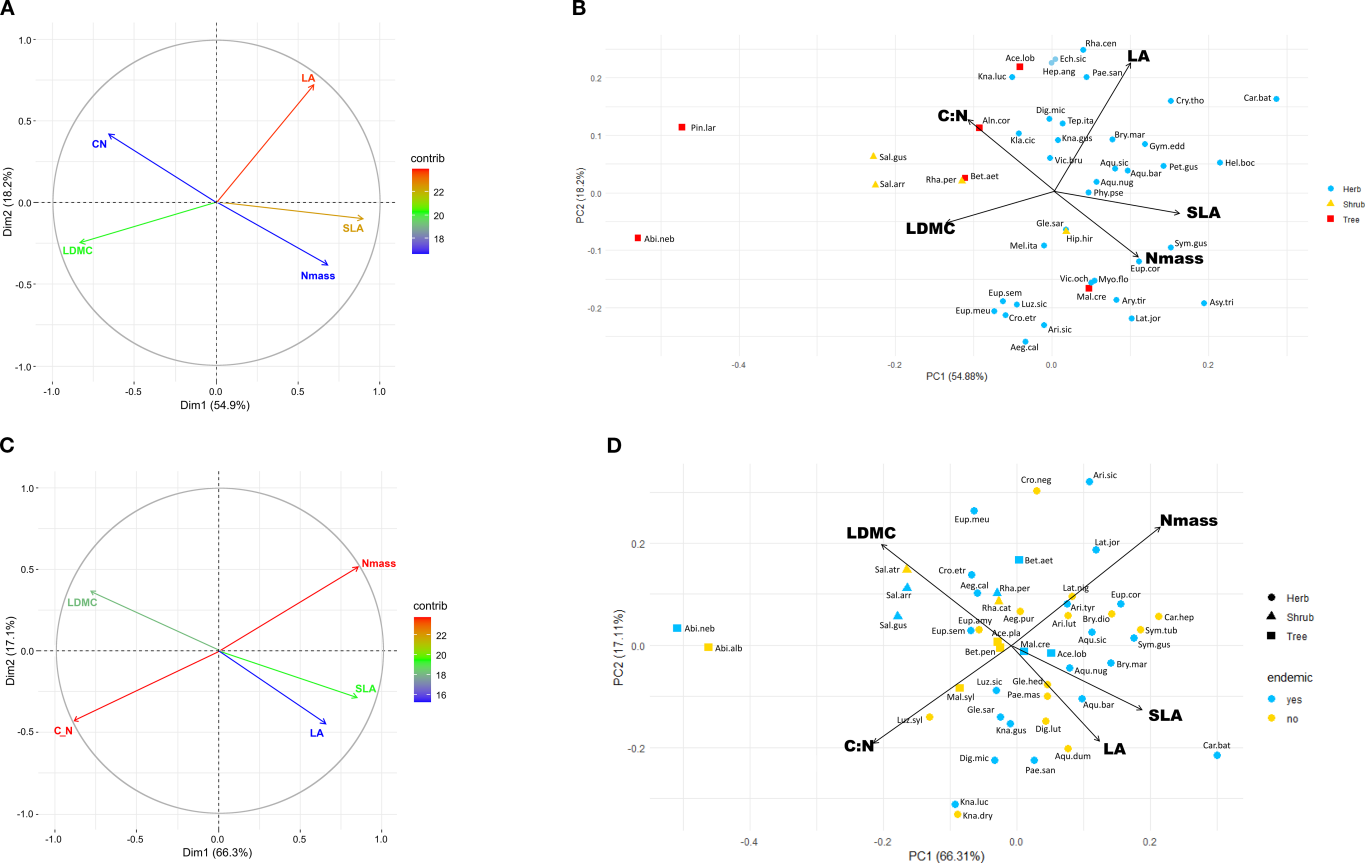
**(A)** Multifactorial analysis showing contributions of LA, SLA, LDMC, Nmass and C:N ratio on the first two ordination axes from the 45 endemics dataset; **(B)** PCA biplot scattergram showing the position of the 45 endemics in the leaf trait space; **(C)** multifactorial analysis of the 27 species pairs dataset; **(D)** PCA biplot scattergram showing distribution of the 27 species pairs in the leaf trait space. Species abbreviations as in **Supplementary Table 1**: Abi.alb, *Abies alba*; Abi.neb, *Abies nebrodensis*; Ace.lob., *Acer cappadocicum* subsp. *lobelii;* Ace.pla., *Acer platanoides;* Aeg.cal., *Aegonychon calabrum;* Aeg.pur., *Aegonychon purpureocaeruleum;* Aeg.pur., *Aegonychon purpureocaeruleum*; Aln.cor., *Alnus cordata*; Aqu.bar., *Aquilegia barbaricina*; Aqu.dum., *Aquilegia dumeticola*; Aqu.nug., *Aquilegia nugorensis*; Aqu.sic., *Aquilegia sicula*; Ari.lut., *Aristolochia lutea*; Ari.sic., *Aristolochia sicula*; Ari.tyr., *Aristolochia tyrrhena*; Asy.tri., *Asyneuma trichocalycinum*; Bet.etn., *Betula etnensis*; Bet.pen., *Betula pendula*; Bry.dio., *Bryonia dioica*; Bry.mar., *Bryonia marmorata*; Car.bat., *Cardamine battagliae*; Car.hep., *Cardamine heptaphylla*; Cro.etr., *Crocus etruscus*; Cro.neg., *Crocus neglectus*; Cry.tho., *Cryptotaenia thomasii*; Dig.lut., *Digitalis lutea*; Dig.mic., *Digitalis micrantha*; Ech.sic., *Echinops siculus*; Eup.amy., *Euphorbia amygdaloides*; Eup.cor., *Euphorbia corallioides*; Eup.meu., *Euphorbia meuselii*; Eup.sem., *Euphorbia semiperfoliata*; Gle.hed., *Glechoma hederacea*; Gle.sar., *Glechoma sardoa*; Gym.sci., *Gymnospermium scipetarum* subsp*. eddae*; Hel.boc., *Helleborus viridis* subsp. *bocconei*; Hep.ang., *Heptaptera angustifolia*; Hyp.hir., *Hypericum hircinum* subsp. *hircinum*; Kla.cic., *Klasea flavescens* subsp. *cichoracea*; Kna.dry., *Knautia drymeja*; Kna.gus., *Knautia gussonei*; Kna.luc., *Knautia lucana*; Lat.jor., *Lathyrus jordanii*; Lat.nig., *Lathyrus niger*; Luz.sic., *Luzula sylvatica* subsp. *sicula*; Luz.syl., *Luzula sylvatica* subsp. *sylvatica*; Mal.cre., *Malus crescimannoi*; Mal.syl., *Malus sylvestris*; Mel.ita., *Melampyrum italicum*; Myo.flo., *Myosotis decumbens* subsp*. florentina*; Pae.mas., *Paeonia mascula*; Pae.san., *Paeonia sandrae*; Pet.gus., *Petagnaea gussonei*; Phy.pse., *Phyteuma ovatum* subsp. *pseudospicatum*; Pin.lar., *Pinus nigra* subsp. *laricio*; Rha.cat., *Rhamnus cathartica*; Rha.per., *Rhamnus persicifolia*; Rha.cen., *Rhaponticoides centaurium*; Sal.arr., *Salix arrigonii*; Sal.atr., *Salix atrocinerea* subsp. *atrocinerea*; Sal.gus., *Salix gussonei*; Sym.gus., *Symphytum gussonei*; Sym.tub., *Symphytum tuberosum*; Tep.ita., *Tephroseris italica*; Vic.bru., *Vicia brulloi*; Vic.och., *Vicia ochroleuca*.

**Figure 2:**
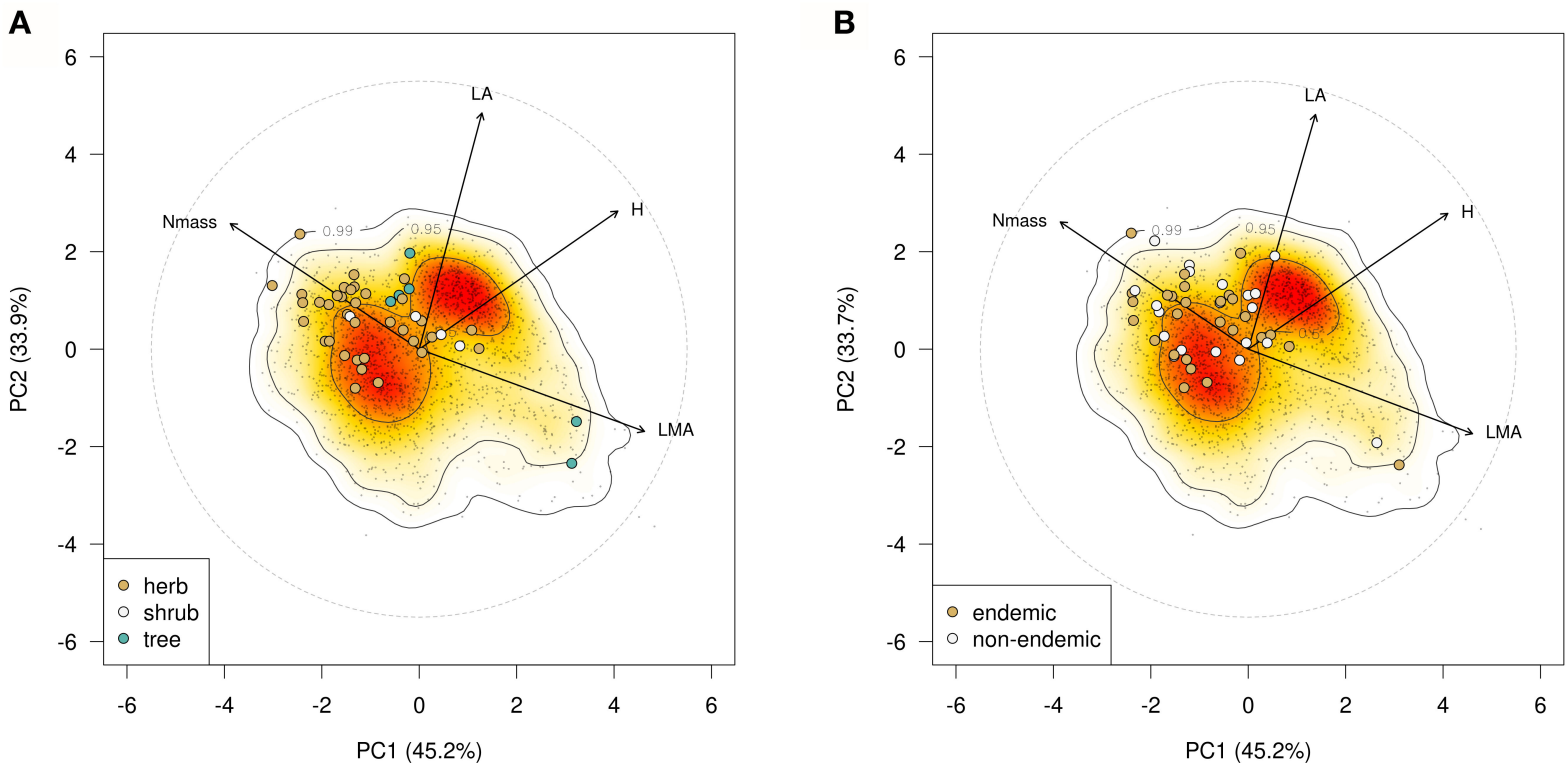
**(A)** Phenospace ordination based on plant height (H), LA, LMA and Nmass showing position of the 45 examined forest endemics within the Global Spectrum of Plant Form and Function of Díaz et al. (2016); **(B)** Phenospace ordination of the 27 endemic-non endemic species pairs.

Multifactorial and PCA analysis of the species means dataset (**Figures 1A, B**) accounted for 73.1% of the total variation, a percentage similar to that observed in the single-leaf dataset (**Supplementary Figure 4**). LA and SLA contributed most strongly to this variation followed by leaf dry matter content (LDMC, 20), while leaf nitrogen content (Nmass) and the C:N ratio contributed less (<18). Herbaceous taxa had tendentially higher scores on the positive side of Dimension 1 (54.2%) associated with LA, SLA and Nmass, while woody species were more widely scattered along the negative side associated with C:N ratio and LDMC; tree species were also widely spaced along Dimension 2 (18.2%). In the Phenospace ordination (**Figure 2A**), endemics were mostly scattered along the Nmass and LMA axes of variation. Many of the herbaceous endemics lied in the high Nmass zone, characterized by a low density of species in the global leaf spectrum (yellow to white areas); similarly, the four endemic angiosperm trees were positioned in the high Nmass zone, outside the high density area of woody species in the global spectrum (orange to red areas). In contrast, the two endemic conifer trees were in the low Nmass and high LMA zone, far from the high density area of woody species (close to the 0.95 density area limit).

**Figure 3:**
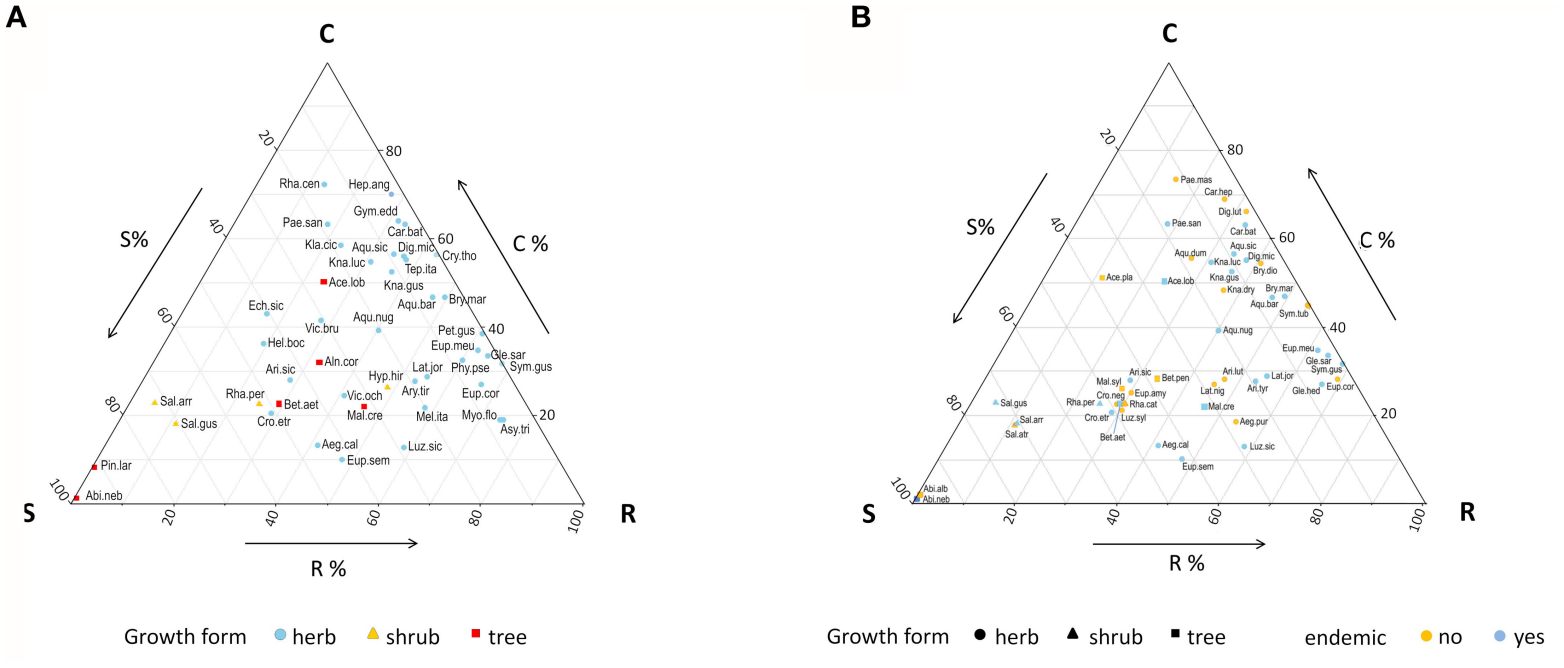
Grime triangles showing **(A)** the position of the 45 forest endemics within the CSR plant strategies scheme, and **(B)** the position of the 27 endemic-non endemic species pairs. Species abbreviations are given in **Supplementary Table 1**.

The endemic taxa were widely spaced in the CSR scheme and with tendentially elevated values on the C and R dimensions (**Figure 3A**). However, these two strategies were dominant in the herbaceous species, while the woody endemics were characterized by higher stress tolerance (**Supplementary Table 3**).

### 3.2 Environmental variables

**Figure 4:**
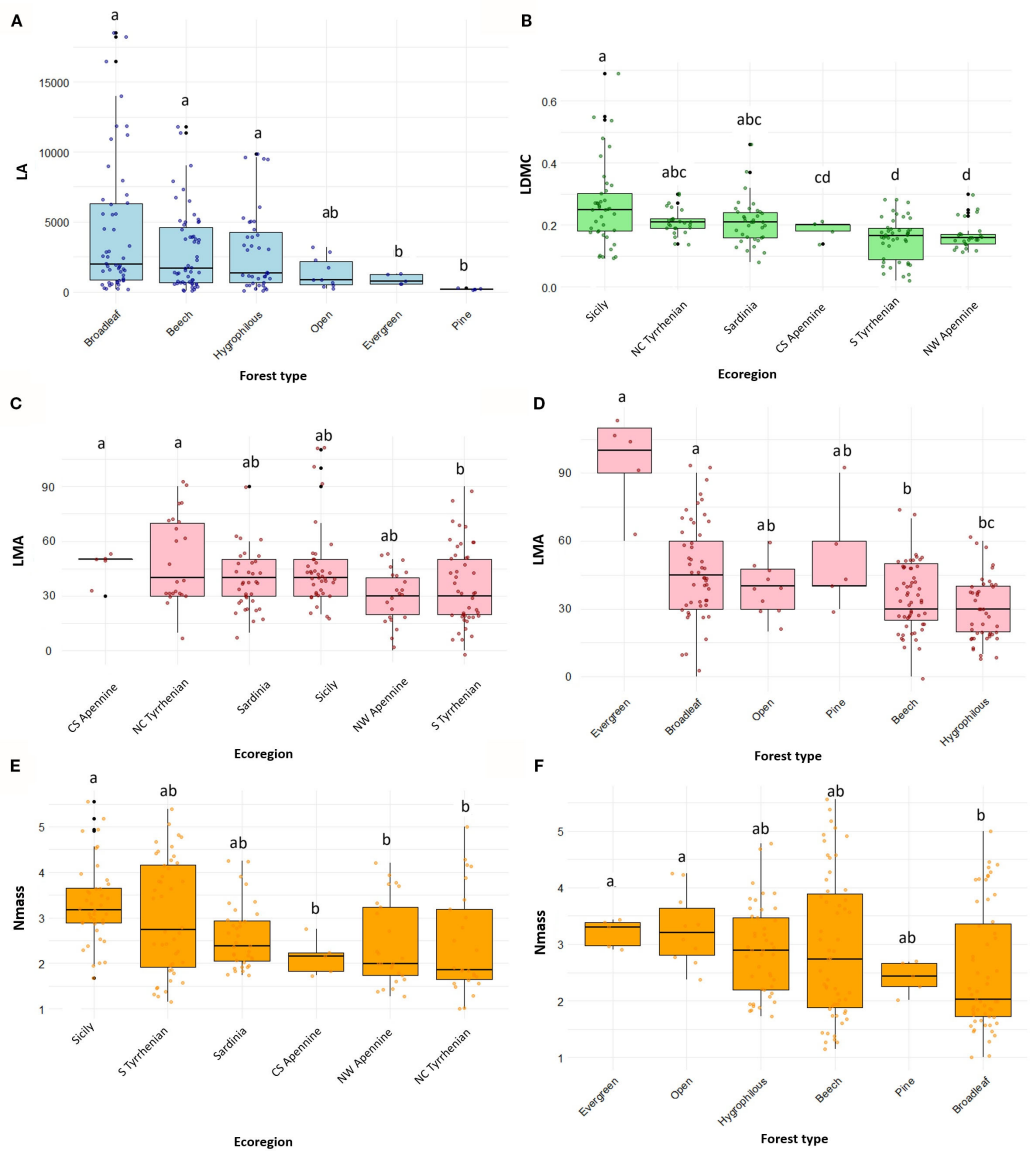
Variation of leaf trait in forest endemics across most significant environmental factors (Forest type and Ecoregion) according to model results (**Table 2**): **(A)** Leaf Area (LA) across forest types **(B)** Leaf Dry Matter Content (LDMC, expressed as g/g) across ecoregions **(C, D)** Leaf Mass per Area (LMA) across ecoregions and forest types, respectively **(E, F)** Nitrogen mass per unit leaf dry mass (Nmass) across ecoregions and forest types, respectively. Boxplots display the median (horizontal line), interquartile range (box), and data dispersion (whiskers and individual points). Forest types and ecoregions are ordered based on decreasing median values of the respective traits. Different letters indicate significant pairwise differences among groups according to *post hoc* tests (Tukey’s HSD with *emmeans* after ANOVA, or Dunn’s test after Kruskal–Wallis, depending on model assumptions).

Mean annual temperature (MAT) significantly reduced LA, while latitude had no significant effect (**Table 2**). Among factorial variables, forest type was selected as the most significant driver for LA; broadleaf, beech and hygrophilous forests had a positive effect on LA, compared with evergreen, open and pine forests (**Figure 4A**). Increasing latitude and precipitation had instead a negative effect on LDMC. This trait was also influenced by ecoregion, highlighting distinct patterns between the insular Tyrrhenian and the Apennine regions, with highest values in Sicily and lowest in the NW Apennine region (**Figure 4B**). LMA exhibited a positive relationship with temperature and was significantly affected also by ecoregion and forest type; endemics from the central and southern Apennines and the northern and central Tyrrhenian regions and evergreen forests tended to have higher LMA values (**Figures 4C, D**). Nmass declined significantly with latitude but increased with precipitation. Ecoregion and forest type were also significant drivers for this trait. Endemics from Sardinia, Sicily and the south Tyrrhenian ecoregion had higher Nmass compared to those from more northern ecoregions (**Figure 4E**). Moreover, endemics from evergreen and open forests exhibited significantly higher Nmass than those from broadleaf forests (**Figure 4F**). Soil type was not found to have significant effects on leaf traits.

**Table 2:** Optimal linear model structures relating leaf traits (LA, LDMC, LMA and Nmass; C:N not shown because no significant results) to environmental variables (latitude, ecoregion, forest type, and tree shade casting ability-SCA in interaction with habitus-W/H).

	LA	LDMC	LMA	Nmass
Continuous variables				
Latitude	↓ 0.09	↓ < 0.001	/	↓ < 0.001
MAT	↓ < 0.001	/	↑ < 0.001	/
MAP	/	↓ < 0.001	/	↑ 0.015
Factor variables				
Ecoregion	/	< 0.001	< 0.001	< 0.001
Forest type	0.021	/	< 0.001	0.022
SCA/WH	0.73	/	/	0.1
R^2^	0.199	0.278	0.20	0.386
Adjusted R^2^	0.155	0.248	0.133	0.332

[R syntax of the starting model: Y ~ Latitude + ecoregion + forest type + soil type + SCA:W/H + MAT + MAP]. For continuous variables (latitude, mean annual temperature-MAT and mean annual precipitation-MAP), the table gives the significance level and the positive (↑) or negative (↓) direction of the effect; for factor variables (ecoregion, forest type, SCA:W/H) only the significance level is reported (soil type not shown because not significant). The direction of significant effects is shown in **Figures 4A-F**. R^2^ refers to the fraction of the variation explained by the model structure, and adjusted R^2^ takes into account the number of independent variables used for predicting the target variable.

### 3.3 Trait variation across species pairs

Results from mixed model analysis of the 27 species pairs dataset (**Table 3**) revealed a significant negative effect of the endemic condition on LA, which was stronger in the herbaceous taxa (**Supplementary Table 4**). Decrease in LA was coupled with a positive effect on LMA, similarly in herbaceous and woody taxa. In the herbaceous species pairs, Nmass was higher in the endemic taxa, while in the woody pairs no differences were detected between endemics and non-endemics. This resulted in an overall weak divergence between endemics and widespread congeners, paralleled by a decrease in the C:N ratio. LDMC did not significantly differ between endemics and non-endemics. Significance of the differences in each trait within each species pair are given in **Supplementary Table 5**.

**Table 3:** Model results of endemic vs non-endemic species pair analysis (n = 27 pairs) showing direction (estimate) and significance of general effects on LA, LDMC, LMA, Nmass and C:N ratio and on C, S and R strategies.

Trait/strategy	Estimate	P-value	R^2^ marginal	R^2^ conditional
LA	-2206.86	***	0.02	0.67
LDMC	-4.518e-03	n.s.	0.01	0.43
LMA	5.082e-03	***	0.01	0.70
Nmass	1.6149	*	0.01	0.67
C:N	-1.3045	**	0.003	0.88
C	-2.8949	***	0.005	0.86
S	-4.1195	***	0.005	0.76
R	7.0058	***	0.03	0.63

Significance level: *p<0.05, **p<0.01, ***p<0.001; n.s. not significant.

In relation to the distribution pattern within pairs, divergence in LA between endemic and non-endemic congeners decreased in the order allopatric > parapatric > sympatric, without significant differences between the latter two groups (**Table 4**). Difference in LMA was also smaller in sympatric pairs than in the other two groups, while for Nmass the mean difference in the allopatric pairs was lower than in the two other groups. The mean difference in C:N was highest in the parapatric pairs, while divergence in LDMC was similar in the three groups.

**Table 4:** Mean value and standard deviation of differences in each trait between endemic and non-endemic congeners in allopatric (n=10 pairs), sympatric (n=9 pairs) and parapatric groups (n=8 pairs); negative differences indicate lower trait values in the endemic taxa; letters indicate statistically significant groups at p < 0.05.

Leaf trait	Allopatric	Parapatric	Sympatric
LA mm^2^	-4301.9 ± 9317.1^a^	-1540.6 ± 8914.8^b^	-998.73 ± 2863.4 ^b^
LDMC g.g^-1^	-0.026 ± 0.091^a^	-0.01 ± 0.20 ^a^	0.003 ± 0.22 ^a^
LMA g.m^-2^	34.4 ± 20.8 ^ab^	69.54 ± 29.8 ^a^	-13.6 ± 33.1 ^b^
Nmass mg.g^-1^	-1.526 ± 6.879 ^a^	4.344 ± 7.271 ^b^	3.108 ± 11.258 ^b^
C:N ratio	0.6284 ± 5.728 ^a^	-4.4083 ± 5.8137 ^b^	-0.890 ± 6.3430 ^a^

Trait variation between endemics and non-endemics translated into differences in terms of CSR strategies. Overall, the C and S dimensions were significantly decreased in the endemics, while the R component was enhanced (**Table 3**; **Figure 3B**); these effects were overall stronger in the herbaceous taxa (**Supplementary Table 4**). Significance of the differences in each trait within each species pair is given in **Supplementary Table 6**.

Multifactorial analysis explained 75.6% of the total variation, with Nmass and C:N ratio contributing most strongly (>22), and SLA and LDMC contributing to a lesser extent (**Figure 1C**); endemics and widespread congeners showed a similar distribution within the trait space. Distance between them was variable depending on the pair, from very low (e.g. *Rhamnus persicifolia-R. cathartica*, *Symphytum tuberosum-S.gussonei*) to relatively high (e.g. *Aquilegia nugorensis-A.dumeticola*, *Cardamine battagliae-C. heptaphylla*).

Phenospace ordination (**Figure 2B**) showed that endemic and non-endemic congeners are closely distributed and mixed to each other, mainly along the Nmass and LMA axes. The trait space explored by the endemics was wider along increasing LMA values but narrower in the plant height axis (H) due to their significantly smaller stature (p < 0.001; not shown).

### 3.4 Trait phylogenetic signal

**Figure 5:**
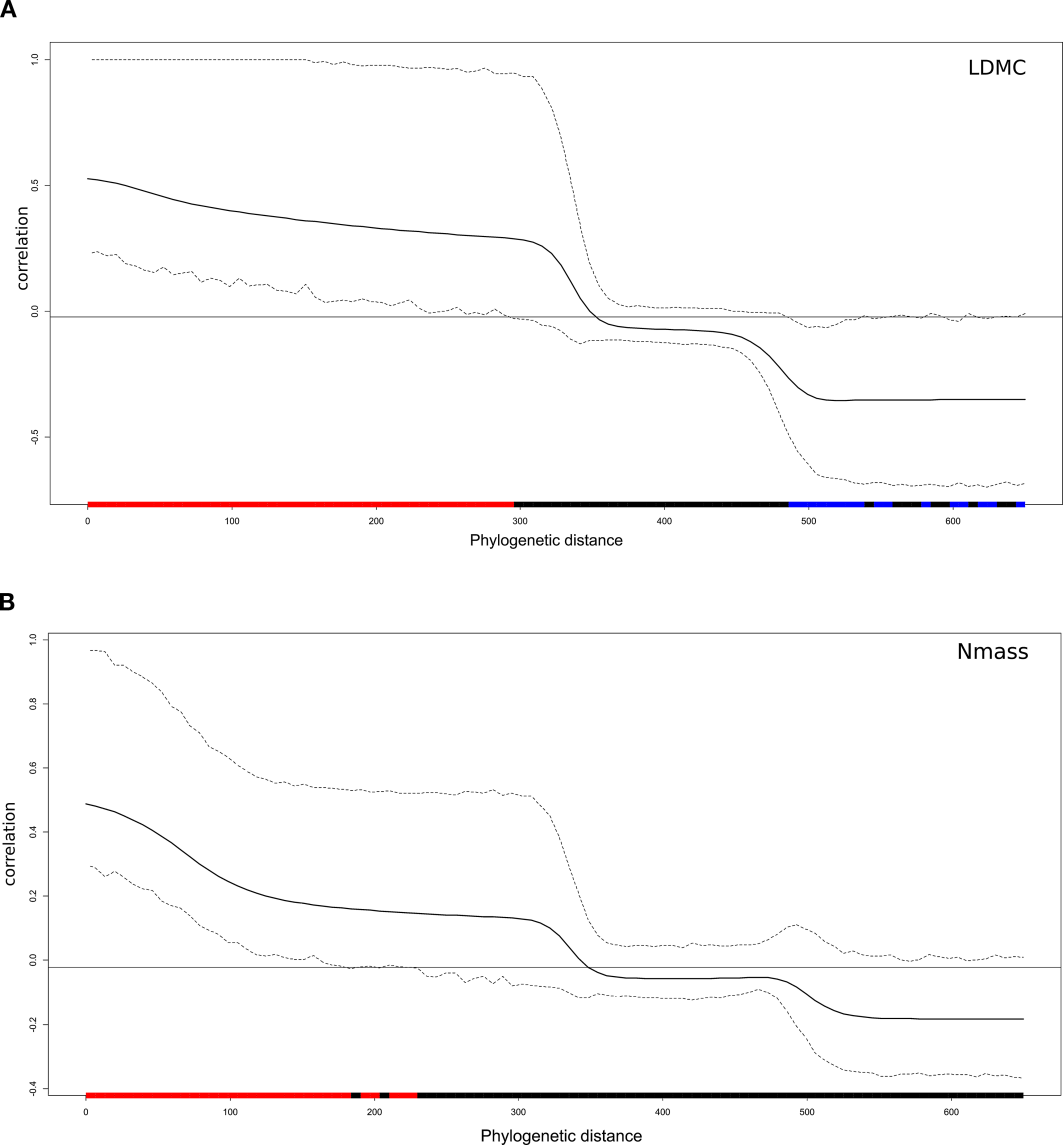
Phylogenetic correlograms of **(A)** LDMC and **(B)** Nmass, showing positive (red bars) and negative (blu bars) correlations at low and high levels of phylogenetic distance between the 45 forest endemics: the horizontal black line indicates the expected value of Moran’s I under the null hypothesis of no phylogenetic autocorrelation.

**Figure 6:**
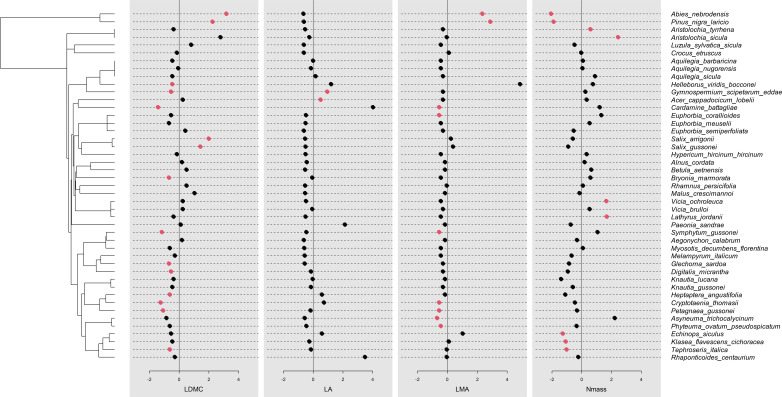
Local Moran’s Index values for each species for LDMC, LA, LMA and Nmass, showing hotspots of phylogenetic autocorrelation; red points indicate significant values, either positive or negative.

Traits displayed a phylogenetic signal of variable intensity, also depending on the metrics used (**Table 5**). Leaf area showed a significant signal based on all three metrics. Pagel’s λ close to 1 pointed to a Brownian model of evolution, while Blomberg K fitted this model less strongly, though still detecting a signal. The Cmean metrics supported a marginally significant autocorrelation between more closely related endemics. The three indices were largely convergent in detecting a signal also for LMA. In the case of LDMC and Nmass, the phylogenetic signal was detected by the λ and Cmean indices, but not K. Overall, Pagel’s λ detected stronger signals in the four traits examined, with values more closely approaching the Brownian model (1). The Nmass correlogram (**Figure 5A**) showed positive autocorrelation for short lags (that is, at low levels of phylogenetic distance), while that of LDMC (**Figure 5B**) showed positive and negative autocorrelation for short and long lags, respectively. In the latter case, therefore, endemics belonging to the same clade tended to share similar trait values while endemics of adjacent clades tended to differ strongly. For LDMC, local hotspots of positive autocorrelation were found in the conifer and in the *Salix* clades, while mainly negative within the Ranunculaceae (*Gymnospermium-Helleborus*), the Apiaceae (*Cryptotaenia, Petagnaea, Heptaptera*) and the Lamiales (*Glechoma, Digitalis*) clades (**Figure 6**). LMA confirmed significant positive autocorrelations in the conifer and mostly negative in the Asterid clade with *Petagnaea, Cryptotaenia*, *Asyneuma* and *Phyteuma* (the latter two taxa in the family Campanulaceae). For Nmass, positive autocorrelation hotspots were detected in the clades of Aristolochiaceae and Fabaceae, while negative in the conifer clade and in the Asteraceae clade with the genera *Echinops, Klasea* and *Tephroseris.*

**Table 5:** Indices of phylogenetic signal and related significance of four leaf traits across 45 Italian forest endemic plants; λ, Pagel’s Lambda; Cmean, Abouheif’s Cmean; K, Bloomberg’s K; p-values determined by 5000 randomizations.

Leaf trait	λ	P-value	Cmean	P-value	K	P-value
LA	0.997	0.001	0.183	0.042	0.363	0.009
LMA	0.628	0.010	0.211	0.029	0.868	0.002
LDMC	0.709	0.001	0.413	0.001	0.121	0.135
Nmass	0.687	0.003	0.307	0.002	0.125	0.053

LA, Leaf Area; LMA, leaf mass per area; LDMC, Leaf Dry Matter Content; Nmass, leaf nitrogen content per unit mass.

## 4 Discussion

A first evidence from this study was the unexpectedly ample variability in the leaf traits across the examined forest endemics, substantially reflecting their diversity in terms of functional types, phylogenetic relationships, biogeographical contexts and forest habitat preferences. Plotting the endemics in the global spectrum based on leaf economic traits and plant height (Díaz et al., 2016) showed their wide and essentially continuous distribution in the functional space mainly defined by Nmass, LA and LMA. As these traits are among those that better reflect the species position along the acquisitive-conservative axis (Garnier et al., 2015), endemics resulted broadly spaced along the resource use gradient, though sharing common adaptive features to the forest macrohabitat such as their shade-tolerant character in the case of understory species. Accordingly, our findings support that the acquisitive-conservative axis emerges even in relatively homogeneous environments, due to the phylogenetic and biogeographic divergence between species (Gorné et al., 2022). Based on the mean values reported at the global scale (Díaz et al., 2022), endemics showed relatively high values of LA and Nmass, while slightly lower for LMA. However, woody and herbaceous taxa showed significant differences in all examined traits, in line with previous findings (e.g. An et al., 2021; Matsuo et al., 2024). The higher LA and Nmass and lower LMA displayed by the herbaceous taxa supported that understory species have more acquisitive traits compared with species in environments with growth limiting factors (Cornelissen et al., 2003; Poorter et al., 2009). Overall, this was supported by the dominance of the C and R components in these taxa, pointing to their relative ability for fast use of resources for growth. Stress tolerance was instead poorly implemented, unlike in other Mediterranean endemics mostly found in open and water-limited habitats (Hand et al., 2017). The latter strategy was more implemented by the woody endemics, which showed more resource-conservative trait values (e.g. lower LA, higher leaf mass per area and lower Nmass), in line with existing evidence for many angiosperms of forest habitats (Matsuo et al., 2024). Stress tolerance was especially implemented in the two endemic conifers with high density needle-like leaves from the Sicilian mountains with nutrient poor soils and intense summer drought (Pignatti et al., 2017).

Leaf area was the most variable trait, varying by 3140-fold among species between *Abies nebrodensis* and the large hemicryptophyte *R. centaurium* (**Supplementary Figure 2A, B**). Leaf area is globally one of the most variable leaf traits (Wright et al., 2017), and dependent on functional groups, clades, biomes and habitats (Garnier et al., 2015; Price et al., 2014; Wright et al., 2017). In the forest endemics examined here, it appeared influenced by phylogenetic relationships, approaching a Brownian model of trait evolution based on Pagel’s λ, as well by environmental variables related to climate and forest habitat. The negative relationship with temperature (MAT) is likely associated with the frequent water limitation of warm areas of the Mediterranean regions, while the cooler conditions of beech forests and other hygrophilous forest communities, especially at lower latitudes, supported larger leaf size in the endemics. Overall, these findings fit global patterns that large-leaved species predominate in humid and warm environments while leaf size decreases in species of warm but arid sites and at higher latitudes (Li et al., 2024; Wright et al., 2017). Remarkably, however, species pairs analysis pointed to a substantial negative effect of the endemic condition on this trait, especially in the herbaceous taxa. Consequent functional differences with respect to widespread congeners from similar forest habitats could be reduced photosynthesis rates as well as effects on leaf water balance and temperature regulation, all directly dependent on LA (Cornelissen et al., 2003; Díaz et al., 2016; Falster and Westoby, 2003). Notably, the divergence in LA resulted positively related to the level of geographic separation between endemics and non-endemic congeners, thus stronger in allopatric than in parapatric and, even more, sympatric species pairs. Allopatric speciation has thus likely contributed to a significant evolutionary divergence in this key trait, likely in concomitance with adaptive processes to ecological constraints in distinct biogeographical regions. Our results are in line with recent evidence that this mode of speciation, more than others, promotes leaf area divergence among allopatric congeneric species living under different habitat conditions (Vargas et al., 2023).

Leaf mass per area varied about ten-fold among the examined endemics and was on average slightly lower than the global mean of 72.4 g m^-2^ reported in Díaz et al. (2022). Considerably higher values occurred in the woody than in the herbaceous taxa, as already known from a previous study (Poorter et al., 2009). Environmental factors and, to some extent, phylogenetic relationships between taxa had a significant influence on LMA, in line with data from a wide range of Mediterranean species investigated by De la Riva et al. (2018). Ecoregion and, even more than for LA, type of forest habitat resulted important drivers of variation, in apparent contrast with previous evidence for remarkably small differences between different types of forest worldwide (Poorter et al., 2009). Mean annual temperature and evergreen forests had both a positive effect on LMA, showing the more resource-conservative behavior of endemics from warmer areas and drought-adapted forests in the southern and central Apennines and the Tyrrhenian regions. This supports general evidence that species with tendentially high LMA are those from habitats where either drought, nutrient limitation or both strongly hamper growth, including more or less open woodlands (Poorter et al., 2009). Also in the forest endemics examined here, therefore, LMA increases with the relative abundance of epidermal and sclerenchyma tissue and with reduced intercellular air spaces in the mesophyll, as an adaptation to life in sites with limited water availability (De la Riva et al., 2016; Fan et al., 2024).

Similarly to LA, divergence in LMA was stronger within pairs formed by allopatric and parapatric taxa than in those including sympatric ones, suggesting the above histo-anatomical adjustments to have originated in concomitance with species range separation and adaptive vicariance.

A relevant finding was the consistent increase of LMA in the endemics, compared with the congeneric species. Combined with reduction in LA and the smaller stature, this clearly points to a lower acquisitive ability and a stronger implementation of a resource conservation attitude than in widespread, closely related taxa. Based on the assumption that LMA is an indicator of the entire plant strategy (Reich, 2014; see also Butrim et al., 2024), the studied endemics appear inherently more inclined to implement a slower return-on investment behavior and higher persistence. When restricting the analysis to plants of forest habitats, therefore, we lend no support to the hypothesis that Mediterranean endemics do not differ from widespread congeners in leaf traits related to resource acquisition or conservation (Hand et al., 2017; Lavergne et al., 2004). Decrease in the C dimension in the endemics also provided evidence for lower resource acquisition ability, though this was associated with a parallel decrease in stress-tolerance and an increase in the “ruderal” component in the herbaceous endemics. The weak increase in Nmass, usually considered as an indicator of plant photosynthetic capacity, respiration and growth rate (Khan et al., 2022; Wright et al., 2004), was in line with the enhanced ruderal strategy. As for LA and LMA, a phylogenetic signal was detected for Nmass, supporting recent findings that aboveground tissue element concentrations in plants, particularly N, Ca, K, and B, is phylogenetically conserved (Furey and Tilman, 2023). Hence, this trait appeared under the complex influence of interacting factors, including latitude and related environmental variables such as precipitation, ecoregion and, to a lesser extent, type of forest habitat. Lower values in the southern endemics from the Sicilian, Sardinian and south Tyrrhenian ecoregions fit evidence that leaf N globally declines with decreasing latitude (Reich and Oleksyn, 2004), though in the present study this increase resulted coupled with precipitation (MAP) and not with temperature (MAT). In addition, the higher Nmass values in endemics from open and evergreen forests support that species in drier or sunnier sites tend to have higher nitrogen concentration in their leaves, likely reflecting a higher photosynthetic efficiency (Fan et al., 2024). Unlike for morpho-functional traits, differences in Nmass were overall larger in parapatric and sympatric species pairs, suggesting this trait to be more influenced by environmental variables, forest habitat and possibly other local site conditions, than by allopatric speciation.

## 5 Conclusions

By providing novel trait values data for 45 endemic taxa of Mediterranean forests and 20 widespread congeners, this work contributes to a better understanding of the functional space exploited by globally rare plants from a major biodiversity hotspot, their resource use attitudes and ecological strategies. Overall, the ample variation in leaf traits across these endemics pointed to a significant differentiation and continuous distribution along the resource acquisitive-conservative gradient defined by LA, LMA and Nmass. Depending on the trait, interspecific variability was influenced by different drivers, including functional group, eco-geographical, climate-related factors and forest habitat, as well as phylogenetic constraints especially for LA and LMA. Herbaceous taxa showed more acquisitive traits increasing resource capture and use efficiency, while the woody taxa were overall more stress-tolerant and with more resource conservative trait values (e.g. higher leaf mass per area). Compared with widespread congeners from similar forest habitats, however, endemics were characterized by lower LA and higher LMA, as well as smaller stature, pointing to their lower acquisitive ability and stronger resource conservation attitude. These features may be involved in their inherently reduced capacity to spread outside their restricted range and often ecological niche over wider territories. Moreover, differences in LA and LMA within allopatric and parapatric pairs were overall larger than in sympatric pairs, suggesting the role of vicariance and range separation in the divergence of morpho-functional leaf traits. Analyzing other traits related to persistence, reproduction and dispersal will further advance our understanding of the functional and ecological space exploited by Mediterranean forest endemic plants, which will help to implement actions for their conservation under the increasing pressures to their habitat.

## Data Availability

The raw data supporting the conclusions of this article will be made available by the authors, without undue reservation.
