# Supplementary material for: Leaf trait variation across Mediterranean forest endemics: drivers and evidence for lower resource acquisition ability than in widespread forest congeners
Source: Front Plant Sci. 2025 Sep 19;16:1664759. doi: 10.3389/fpls.2025.1664759 (PMC12492640; doi:10.3389/fpls.2025.1664759)
Supplement: Supplementary file 1 [file DataSheet1.docx]

Supplementary Material

## Supplementary Figures.

**Supplementary Figure 1.** Sampling sites where endemic and non-endemic species were collected in Italy. More than one species were sampled in part of the sites.


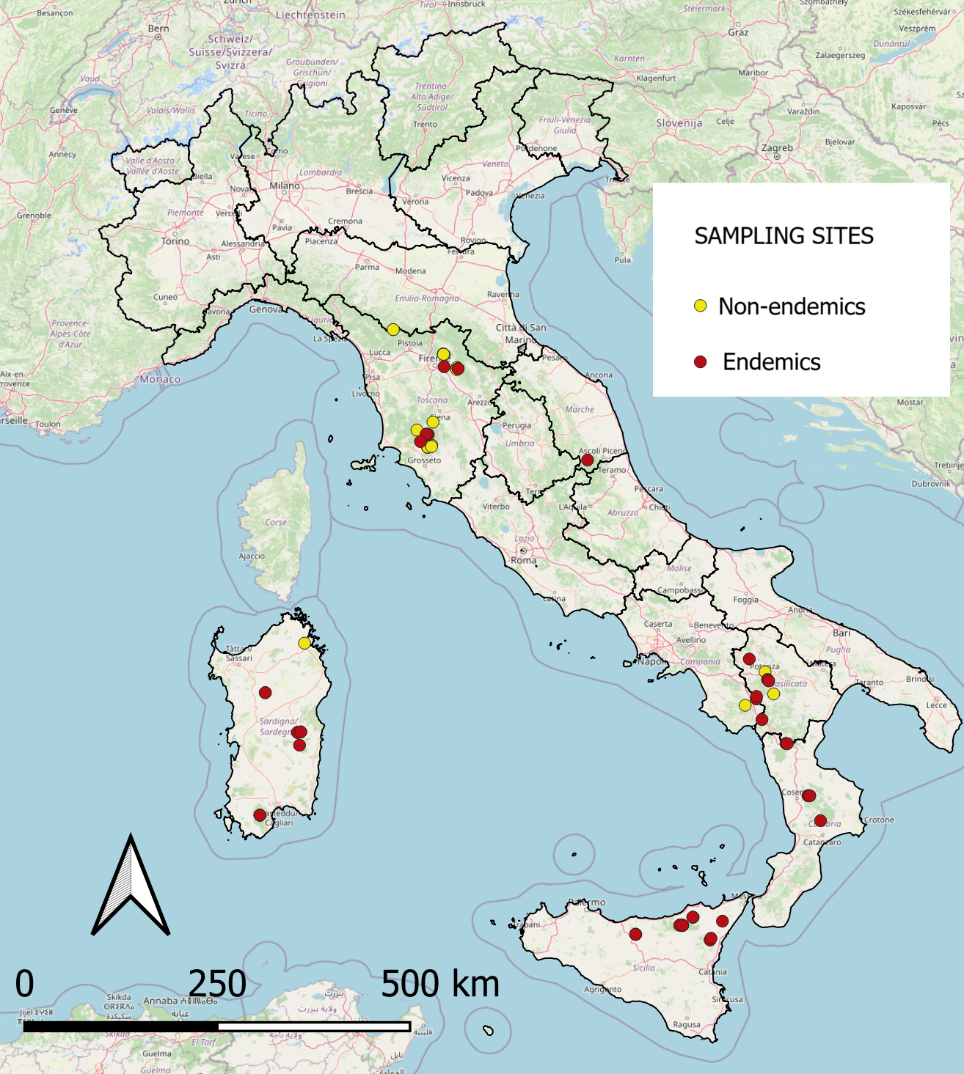


**Supplementary Figure 2.**  Field photos of leaves of twenty Italian endemic forest plants examined in this study. A) *Abies nebrodensis*, B) *Aristolochia sicula* (photo E. Carrari), C) *Aristolochia tyrrhena*, D) *Cardamine battagliae*, E) *Vicia brulloi*, F) *Glechoma sardoa*, G) *Aegonychon calabrum* (photo A. Coppi), H) *Cryptotaenia thomasii*, I) *Euphorbia corallioides*, J) *Euphorbia meuselii*, K) *Crocus etruscus*, L) *Gymnospermium scipetarum* subsp. *eddae*, M) *Acer cappadocicum* subsp. *lobelii*, N) *Rhaponticoides centaurium*, O) *Rhamnus persicifolia* (photo E. Farris), P) *Tephroseris italica*, Q) *Digitalis micrantha*, R) *Asyneuma trichocalycinum*, S) *Petagnaea gussonei*, T) *Symphytum gussonei*. All photos by F. Selvi, except when differently indicated.


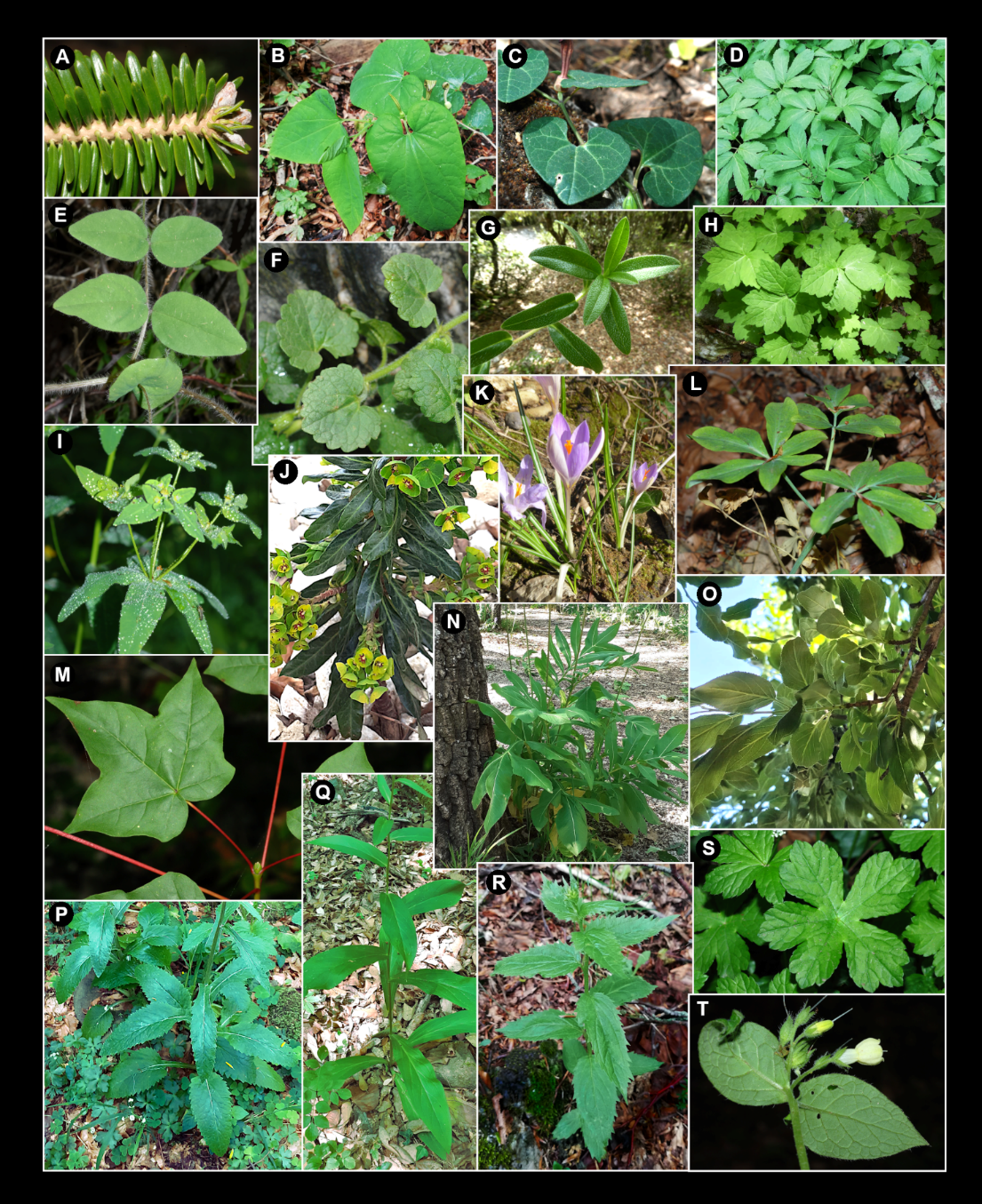


**Supplementary Figure 3.** Circular phylogenetic tree of the 65 forest taxa (45 endemic, 20 non-endemic) investigated in this study.


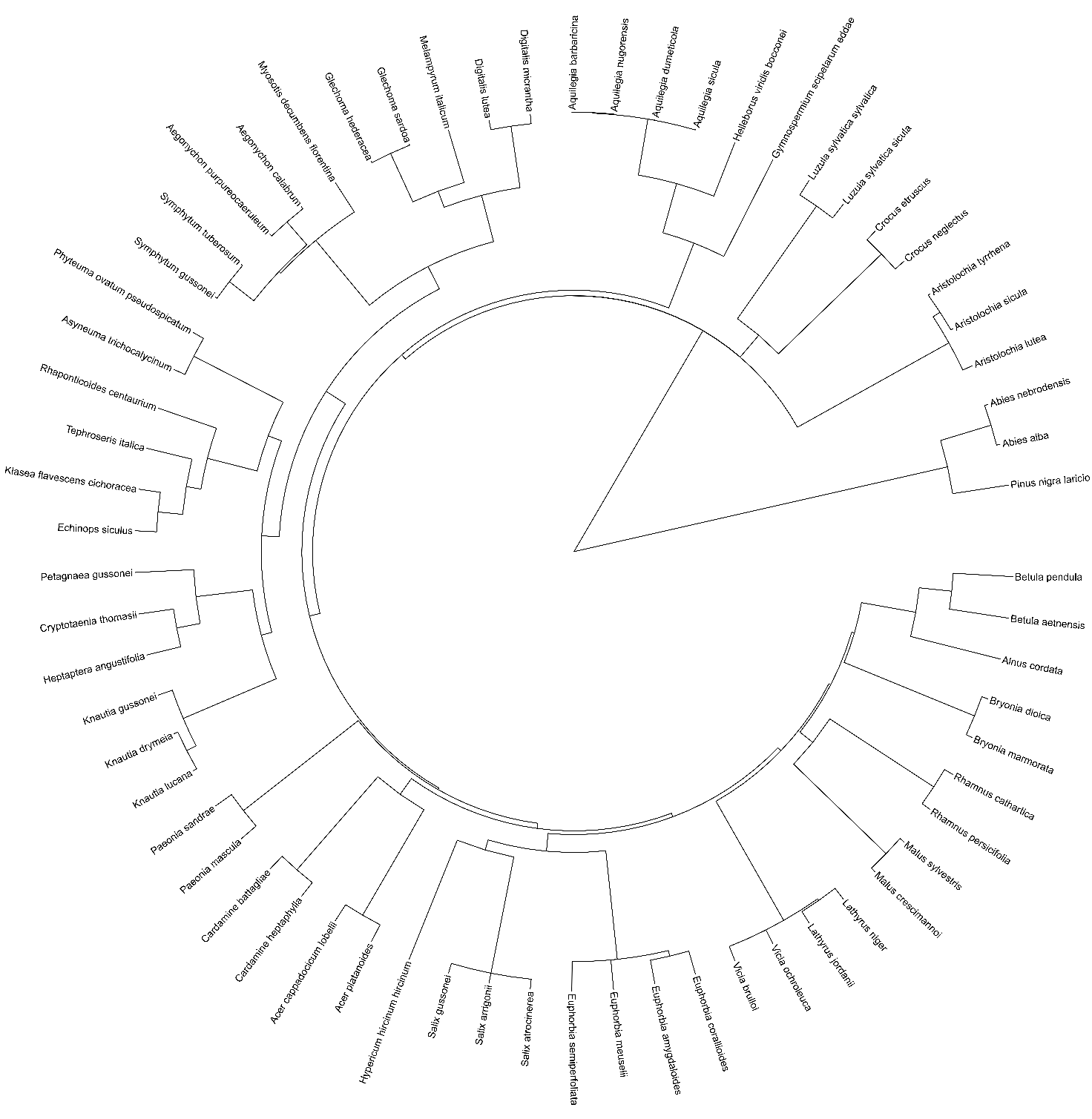


**Supplementary Figure 4**. Trait correlogram.


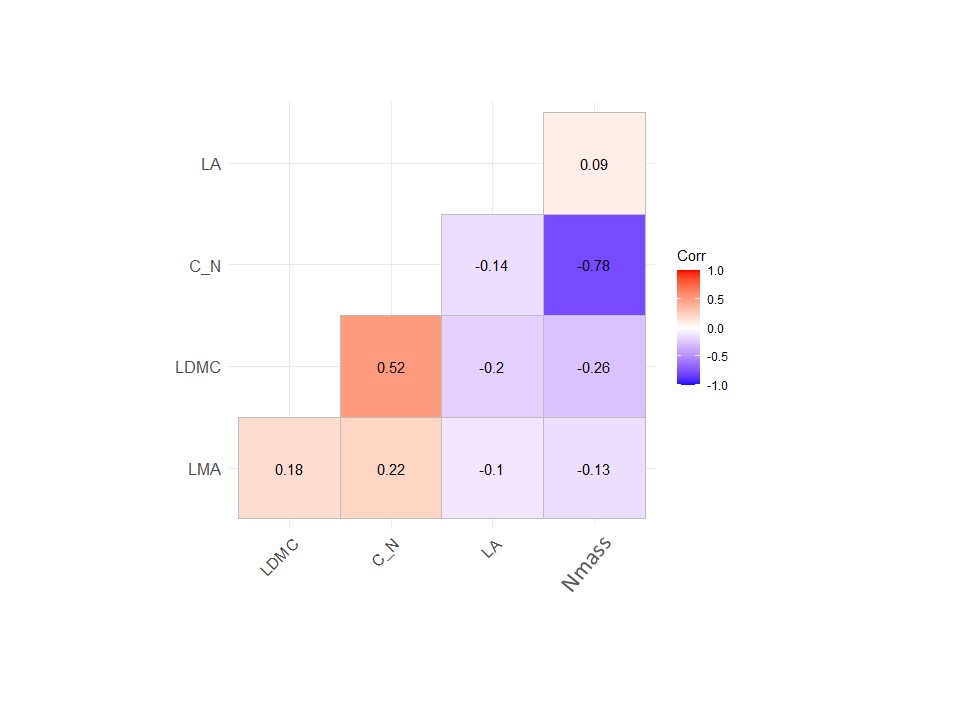


**Supplementary Figure 5**. Scattergram from multifactorial PCA analysis of the single-leaf dataset.


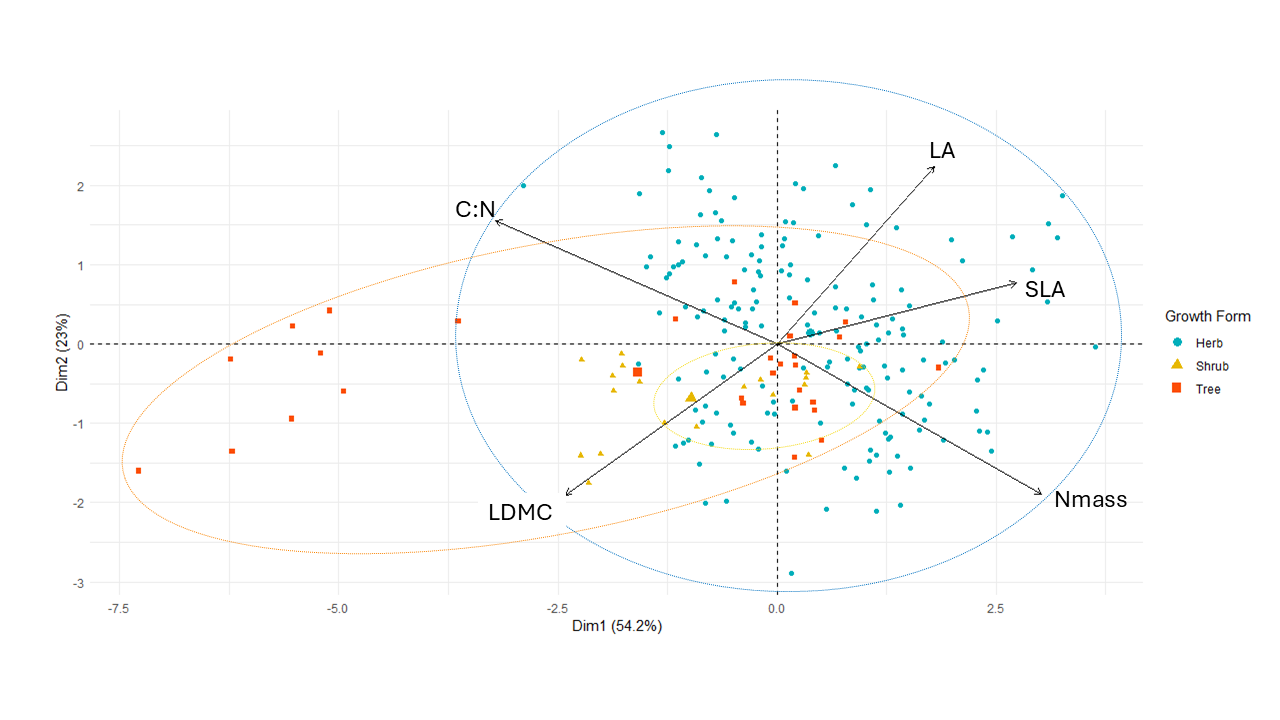


## Supplementary Tables

# Supplementary Table 1. List of the 65 endemic (E) and non-endemic taxa analyzed for plant traits, with species code, family, Raunkiaer’s life form, general distribution (chorotype) of the non-endemic taxa, and geographic origin (Lat and Long N) of the sampled populations; plant nomenclature follows Bartolucci et al. (2024); Italian administrative regions (Reg) are abbreviated.

| Taxon | Code | Family | Chorotype | Life  form | Lat  N | Lon  E | Reg |
| --- | --- | --- | --- | --- | --- | --- | --- |
| Abies alba Mill. | Abi.alb | Pinaceae | CS Eur | P scap | 40.4124 | 15.9253 | Bas |
| Abies nebrodensis (Lojac.) Mattei | Abi.neb | Pinaceae | E | P scap | 37.8399 | 14.0243 | Sic |
| Acer cappadocicum subsp. lobelii (Ten.) Murray | Ace.lob | Sapindaceae | E | P scap | 40.7779 | 15.5892 | Bas |
| Acer platanoides L. | Ace.pla | Sapindaceae | Eur-Caucas | P scap | 43.7522 | 11.5602 | Tos |
| Aegonychon calabrum (Ten.) Holub | Aeg.cal | Boraginaceae | E | H scap | 39.0691 | 16.5706 | Cal |
| Aegonychon purpureocaeruleum (L.) Holub | Aeg.pur | Boraginaceae | S Eur-Pontic | H scap | 42.9529 | 11.1570 | Tos |
| Alnus cordata (Loisel.) Duby | Aln.cor | Betulaceae | E | P scap | 39.8901 | 16.0945 | Cal |
| Aquilegia barbaricina Arrigoni & E.Nardi | Aqu.bar | Ranunculaceae | E | H scap | 40.0129 | 9.4085 | Sar |
| Aquilegia dumeticola Jord. | Aqu.dum | Ranunculaceae | SE Eur | H scap | 43.7320 | 11.5557 | Tos |
| Aquilegia nugorensis Arrigoni & E.Nardi | Aqu.nug | Ranunculaceae | E | H scap | 39.8721 | 9.3918 | Sar |
| Aquilegia sicula (Strobl) E.Nardi | Aqu.sic | Ranunculaceae | E | H scap | 37.9393 | 14.6708 | Sic |
| Aristolochia lutea Desf. | Ari.lut | Aristolochiaceae | CE Eur-Medit | G bulb | 42.9736 | 11.2141 | Tos |
| Aristolochia sicula Tineo | Ari.sic | Aristolochiaceae | E | G bulb | 37.9414 | 14.6307 | Sic |
| Aristolochia tyrrhena E.Nardi & Arrigoni | Ari.tyr | Aristolochiaceae | E | G bulb | 40.0071 | 9.3635 | Sar |
| Asyneuma trichocalycinum (Ten.) K.Malý | Asy.tri | Campanulaceae | E | H scap | 39.3362 | 16.3970 | Cal |
| Betula etnensis Raf. | Bet.aet | Betulaceae | E | P scap | 37.7787 | 15.0491 | Sic |
| Betula pendula Roth | Bet.pen | Betulaceae | Eurosib | P scap | 40.2932 | 15.5330 | Sic |
| Bryonia dioica Jacq. | Bry.dio | Cucurbitaceae | Euri-Medit | G rhiz | 40.6439 | 15.8067 | Bas |
| Bryonia marmorata E.Petit | Bry.mar | Cucurbitaceae | E | G rhiz | 40.0071 | 9.3635 | Sar |
| Cardamine battagliae Cesca & Peruzzi | Car.bat | Brassicaceae | E | G rhiz | 39.3353 | 16.4219 | Cal |
| Cardamine heptaphylla (Vill.) O.E.Schulz | Car.hep | Brassicaceae | SW Eur | G rhiz | 43.8836 | 11.3751 | Tos |
| Crocus etruscus Parl. | Cro.etr | Iridaceae | E | G bulb | 42.9527 | 11.1579 | Tos |
| Crocus neglectus Peruzzi & Carta | Cro.neg | Iridaceae | Euri-Medit | G bulb | 43.1256 | 11.0093 | Tos |
| Cryptotaenia thomasii (Ten). DC. | Cry.tho | Apiaceae | E | H scap | 39.8879 | 16.1125 | Cal |
| Digitalis lutea L. | Dig.lut | Plantaginaceae | W Eur | H scap | 44.1283 | 10.6831 | Tos |
| Digitalis micrantha Roth | Dig.mic | Plantaginaceae | E | H scap | 43.8873 | 11.3715 | Tos |
| Echinops siculus Strobl | Ech.sic | Asteraceae | E | H scap | 43.0811 | 11.1658 | Tos |
| Euphorbia amygdaloides L. | Eup.amy | Euphorbiaceae | C Eur-Caucas | Ch suffr | 42.9526 | 11.1564 | Tos |
| Euphorbia corallioides L. | Eup.cor | Euphorbiaceae | E | G rhiz | 39.3353 | 16.4219 | Cal |
| Euphorbia meuselii Geltm. | Eup.meu1 | Euphorbiaceae | E | Ch suffr | 37.8420 | 14.0232 | Sic |
| Euphorbia semiperfoliata Viv. | Eup.sem | Euphorbiaceae | E | H scap | 39.1274 | 8.8457 | Sar |
| Glechoma hederacea L. | Gle.hed | Lamiaceae | Circumbor | H scap | 43.0834 | 11.1393 | Tos |
| Glechoma sardoa (Bég.) Bég. | Gle.sar | Lamiaceae | E | H scap | 40.4258 | 8.9201 | Sar |
| Gymnospermium scipetarum Paparisto & Qosja ex E.Mayer & Pulević subsp. eddae Rosati et al. | Gym.edd | Berberidaceae | E | G bulb | 40.3596 | 15.6851 | Cam |
| Helleborus viridis L. subsp. bocconei (Ten.) Peruzzi | Hel.boc | Ranunculaceae | E | G rhiz | 43.7594 | 11.3842 | Tos |
| Heptaptera angustifolia (Bertol.) Tutin | Hep.ang | Apiaceae | E | H scap | 40.5641 | 15.8397 | Bas |
| Hypericum hircinum L. subsp. hircinum | Hyp.hir | Hypericaceae | E | NP | 38.0274 | 14.8151 | Sic |
| Klasea flavescens (L.) Holub subsp. cichoracea (L.) Greuter & Wagenitz | Kla.cic | Asteraceae | E | H scap | 42.9602 | 11.2055 | Tos |
| Knautia drymeja Heuff. | Kna.dry | Dipsacaceae | SE Eur | H scap | 44.1283 | 10.6831 | Tos |
| Knautia gussonei Szabó | Kna.gus | Dipsacaceae | E | H scap | 42.8226 | 13.3612 | Mar |
| Knautia lucana Lacaita & Szabó | Kna.luc | Dipsacaceae | E | H scap | 40.3863 | 15.6888 | Bas |
| Lathyrus jordanii (Ten.) Ces., Pass. & Gibelli | Lat.jor | Fabaceae | E | G rhiz | 40.1424 | 15.7639 | Bas |
| Lathyrus niger (L.) Bernh. | Lat.nig | Fabaceae | Eur-Caucas | H scap | 42.9530 | 11.1577 | Tos |
| Luzula sylvatica (Huds.) Gaudin subsp. sicula (Parl.) K.Richt. | Luz.sic | Juncaceae | E | H caesp | 37.9393 | 14.6708 | Cal |
| Luzula sylvatica (Huds.) Gaudin subsp. sylvatica | Luz.syl | Juncaceae | SE Eur | H caesp | 43.8798 | 11.3851 | Tos |
| Malus crescimannoi Raimondo | Mal.cre | Rosaceae | E | P scap | 37.9414 | 14.6307 | Sic |
| Malus sylvestris (L.) Mill. | Mal.syl | Rosaceae | C Eur-Caucas | P scap | 42.9596 | 11.2103 | Tos |
| Melampyrum italicum (Beauv.) Soó | Mel.ita | Orobanchaceae | E | T scap | 43.0848 | 11.1377 | Tos |
| Myosotis decumbens Host subsp. florentina Grau | Myo.flo | Boraginaceae | E | H scap | 43.7409 | 11.5761 | Tos |
| Paeonia mascula (L.) Mill. | Pae.mas | Paeoniaceae | Eur-Caucas | G rhiz | 37.8421 | 14.0233 | Sic |
| Paeonia sandrae Camarda | Pae.san | Paeoniaceae | E | G rhiz | 40.4258 | 8.9201 | Sar |
| Petagnaea gussonei (Spreng.) Rauschert | Pet.gus | Apiaceae | E | G rhiz | 38.0270 | 14.8093 | Sic |
| Phyteuma ovatum Honck. subsp. pseudospicatum Pignatti | Phy.pse | Campanulaceae | E | H scap | 43.7409 | 11.5761 | Tos |
| Pinus nigra J.F.Arnold subsp. laricio Palib. ex Maire | Pin.lar | Pinaceae | E | P scap | 37.7987 | 15.0617 | Sic |
| Rhamnus cathartica L. | Rha.cat | Rhamnaceae | S Eur-Pontic | P caesp | 43.2053 | 11.2301 | Tos |
| Rhamnus persicifolia Moris | Rha.per | Rhamnaceae | E | P caesp | 40.0124 | 9.4095 | Sar |
| Rhaponticoides centaurium (L.) M.V.Agab. & Greuter | Rha.cen | Asteraceae | E | H scap | 40.5487 | 15.8568 | Bas |
| Salix arrigonii Brullo | Sal.arr | Salicaceae | E | P caesp | 39.1274 | 8.8456 | Sar |
| Salix atrocinerea Brot. subsp. atrocinerea | Sal.atr | Salicaceae | W Medit Atl | P caesp | 40.9433 | 9.4607 | Sar |
| Salix gussonei Brullo & Spamp. | Sal.gus | Salicaceae | E | P caesp | 39.1274 | 8.8457 | Sic |
| Symphytum gussonei F.W.Schultz | Sym.gus | Boraginaceae | E | G rhiz | 37.9393 | 14.6708 | Sic |
| Symphytum tuberosum L. | Sym.tub | Boraginaceae | SE Eur | G rhiz | 43.8836 | 11.3751 | Tos |
| Tephroseris italica Holub | Tep.ita | Asteraceae | E | H scap | 43.7393 | 11.5771 | Tos |
| Vicia brulloi Sciandr., Giusso, Salmeri & Miniss. | Vic.bru | Fabaceae | E | H scap | 37.9845 | 15.2194 | Sic |
| Vicia ochroleuca Ten. | Vic.och | Fabaceae | E | H scap | 43.0101 | 11.0576 | Tos |

**Supplementary Table 2**. Elevation, ecoregion, main forest type, dominant tree species, corrected shade casting ability (SCA), main soil type, mean annual temperature (MAT) and precipitation (MAP) for each endemic taxon/population sampled (species codes in Supplementary Table 1); see Materials and Methods for explanation of the variables.

| Taxon  (code) | Elev.  (m) | | Ecoregion | | Forest type | Dominant tree | SCA | Soil type | MAT  (°C) | MAP  (mm) |
| --- | --- | --- | --- | --- | --- | --- | --- | --- | --- | --- |
| Abi.neb | | 1560 | | Sicily | Open | − | − | calcareous | 9.65 | 840 |
| Ace.lob | | 1360 | | S Tyrrhenian | Broadleaf | − | − | siliceous | 9.05 | 760 |
| Aeg.cal | | 925 | | S Tyrrhenian | Pine | Pinus nigra | 1 | siliceous | 9.75 | 1337 |
| Aln.cor | | 1215 | | S Tyrrhenian | Broadleaf | − | − | calcareous | 9.85 | 1102 |
| Aqu.bar | | 894 | | Sardinia | Hygrophilous | Alnus glutinosa | 3 | calcareous | 11.55 | 810 |
| Aqu.nug | | 886 | | Sardinia | Hygrophilous | Alnus glutinosa | 3 | calcareous | 12.35 | 806 |
| Aqu.sic | | 1450 | | Sicily | Beech | Fagus sylvatica | 2.5 | calcareous | 9.75 | 964 |
| Ari.sic | | 1250 | | Sicily | Beech | Fagus sylvatica | 5 | calcareous | 10.95 | 886 |
| Ari.tyr | | 1117 | | Sardinia | Open | Pistacia lentiscus | 1 | calcareous | 9.95 | 898 |
| Asy.tri | | 1600 | | S Tyrrhenian | Beech | Fagus sylvatica | 5 | siliceous | 7.85 | 1448 |
| Bet.aet | | 1720 | | Sicily | Open | − | − | basalt | 7.55 | 1182 |
| Bry.mar | | 84 | | Sardinia | Open | Pistacia lentiscus | 1 | siliceous | 9.95 | 898 |
| Car.bat | | 1350 | | S Tyrrhenian | Hygrophilous | Fagus sylvatica | 5 | siliceous | 9.15 | 1453 |
| Cro.etr | | 85 | | NC Tyrrhenian | Broadleaf | Quercs cerris | 3 | siliceous | 15.05 | 657 |
| Cry.tho | | 1100 | | S Tyrrhenian | Hygrophilous | Fagus sylvatica | 5 | calcareous | 10.15 | 1061 |
| Dig.mic | | 590 | | NW Apennine | Broadleaf | Castanea sativa | 3 | siliceous | 11.35 | 1439 |
| Ech.sic | | 310 | | NC Tyrrhenian | Broadleaf | Quercus cerris | 1.5 | siliceous | 13.55 | 904 |
| Eup.cor | | 1350 | | S Tyrrhenian | Hygrophilous | Alnus glutinosa | 3 | siliceous | 9.15 | 1453 |
| Eup.meu1 | | 1560 | | Sicily | Evergreen | Quercus ilex | 2.5 | calcareous | 9.65 | 840 |
| Eup.sem | | 373 | | Sardinia | Hygrophilous | Alnus glutinosa | 3 | siliceous | 15.35 | 695 |
| Gle.sar | | 750 | | Sardinia | Hygrophilous | Alnus glutinosa | 3 | basalt | 12.85 | 592 |
| Gym.edd | | 1165 | | S Tyrrhenian | Broadleaf | Quercus cerris | 5 | calcareous | 9.65 | 883 |
| Hel.boc | | 350 | | NW Apennine | Broadleaf | Querc pubescens | 3 | siliceous | 13.05 | 1289 |
| Hep.ang | | 1118 | | S Tyrrhenian | Broadleaf | Quercus cerris | 3 | calcareous | 9.95 | 707 |
| Hyp.hir | | 620 | | Sicily | Hygrophilous | Alnus glutinosa | 1.5 | siliceous | 14.55 | 784 |
| Kla.cic | | 120 | | NC Tyrrhenian | Broadleaf | Quercus cerris | 1.5 | calcareous | 14.75 | 706 |
| Kna.gus | | 1332 | | CS Apennine | Beech | Fagus sylvatica | 2.5 | siliceous | 7.85 | 1173 |
| Kna.luc | | 1080 | | S Tyrrhenian | Beech | Fagus sylvatica | 2.5 | calcareous | 9.65 | 919 |
| Lat.jor | | 900 | | S Tyrrhenian | Broadleaf | Castanea sativa | 3 | calcareous | 11.25 | 1074 |
| Luz.sic | | 1460 | | Sicily | Beech | Fagus sylvatica | 5 | calcareous | 9.75 | 964 |
| Mal.cre | | 1250 | | Sicily | Beech | Fagus sylvatica | 5 | calcareous | 10.95 | 886 |
| Mel.ita | | 315 | | NC Tyrrhenian | Broadleaf | Quercus cerris | 3 | calcareous | 13.35 | 925 |
| Myo.flo | | 1290 | | NW Apennine | Beech | Fagus sylvatica | 5 | siliceous | 7.45 | 1439 |
| Pae.san | | 750 | | Sardinia | Broadleaf | Quercus pubescens | 3 | basalt | 12.85 | 592 |
| Pet.gus | | 680 | | Sicily | Hygrophilous | Alnus glutinosa | 3 | siliceous | 14.55 | 784 |
| Phy.pse | | 1290 | | NW Apennine | Beech | Fagus sylvatica | 5 | siliceous | 7.45 | 1439 |
| Pin.lar | | 1535 | | Sicily | Open | − | − | basalt | 8.95 | 1094 |
| Rha.per | | 1200 | | Sardinia | Open | − | − | siliceous | 11.55 | 810 |
| Rha.cen | | 1120 | | S Tyrrhenian | Broadleaf | Quercus pubescens | 1.5 | calcareous | 10.25 | 689 |
| Sal.arr | | 373 | | Sardinia | Open | − | − | siliceous | 15.35 | 695 |
| Sal.gus | | 395 | | Sicily | Open | − | − | basalt | 15.95 | 743 |
| Sym.gus | | 1450 | | Sicily | Beech | Fagus sylvatica | 5 | calcareous | 9.75 | 964 |
| Tep.ita | | 1335 | | NW Apennine | Beech | Fagus sylvatica | 5 | arenaria | 7.45 | 1439 |
| Vic.bru | | 540 | | Sicily | Hygrophilous | Platanus orientalis | 3 | siliceous | 14.85 | 928 |
| Vic.och | | 360 | | NC Tyrrhenian | Broadleaf | Quercus cerris | 3 | calcareous | 13.35 | 725 |

**Supplementary Table 3**. Species mean values and standard deviations for LA, LDMC, LMA, Nmass and C:N ratio of the 45 forest endemic taxa. Mean values of woody and herbaceous taxa are also given, with significance of the differences (*** p < 0.001; ** p< 0.01; * p < 0.05). Mean percentages of Competitive ability (C%), Stress-tolerance (S%) and Ruderality (R%; standard deviations omitted for brevity); species codes in Supplementary Table 1.

| Taxon | LA  mm^2^ | LDMC  g.g^-1^ | LMA g.m^2^ | Nmass,  mg.g^-1^ | C:N | C% | S% | R% |
| --- | --- | --- | --- | --- | --- | --- | --- | --- |
| Abi.neb | 34.7 ± 4.5 | 0.59 ± 0.31 | 241.7 ± 92.4 | 7.39 ± 1.06 | 64.3 ± 8.28 | 1.2 | 98.9 | 0 |
| Ace.lob | 8539.8 ± 2545.2 | 0.27 ± 0.04 | 41.8 ± 9.6 | 30.36 ± 8.44 | 15.17 ± 3.38 | 50.2 | 25.7 | 24.1 |
| Aeg.cal | 233.85 ± 47.7 | 0.26 ± 0.07 | 51.6 ± 15.5 | 24.08 ± 2.89 | 16.72 ± 1.71 | 13.3 | 45.3 | 41.4 |
| Aln.cor | 1707.7 ± 562.3 | 0.26 ± 0.03 | 46.3 ± 5.2 | 29.03 ± 2.1 | 16.28 ± 1.15 | 32.0 | 35.6 | 32.4 |
| Aqu.bar | 4731.1 ± 1731 | 0.18 ± 0.04 | 26.2 ± 8.2 | 27.81 ± 8.14 | 14.95 ± 4.21 | 46.7 | 6.4 | 47.0 |
| Aqu.nug | 3738.3 ± 1526.7 | 0.23 ± 0.09 | 31.9 ± 13.7 | 27.43 ± 2.1 | 13.69 ± 0.85 | 39.3 | 20.5 | 40.2 |
| Aqu.sic | 5927.7 ± 3238.1 | 0.18 ± 0.04 | 42.0 ± 13.1 | 35.61 ± 5.56 | 12.14 ± 2.06 | 56.5 | 8.9 | 34.6 |
| Ari.sic | 2908 ± 1417.7 | 0.43 ± 0.21 | 58.4 ± 28.7 | 50.32 ± 3.67 | 8.67 ± 0.61 | 28.1 | 43.5 | 28.4 |
| Ari.tyr | 905.45 ± 506.5 | 0.19 ± 0.03 | 38.2 ± 14.6 | 32.74 ± 7.7 | 12.56 ± 2.6 | 27.7 | 19.2 | 53.1 |
| Asy.tri | 748.3 ± 125.0 | 0.13 ± 0.06 | 14.6 ± 5.3 | 47.52 ± 4.25 | 8.66 ± 1.24 | 19.1 | 6.3 | 74.6 |
| Bet.aet | 945.9 ± 190.2 | 0.3 ± 0.03 | 48.7 ± 4.1 | 33.09 ± 3.33 | 13.78 ± 1.9 | 22.6 | 48.2 | 29.3 |
| Bry.mar | 4354.5 ± 2589.5 | 0.15 ± 0.04 | 27.7 ± 8.9 | 32.66 ± 5.77 | 11.86 ± 1.65 | 46.9 | 3.8 | 49.4 |
| Car.bat | 34197.63 ± 14920.5 | 0.06 ± 0.02 | 15.6 ± 11.7 | 38.57 ± 5.27 | 10.85 ± 1.17 | 63.2 | 3.3 | 33.4 |
| Cro.etr | 232.05 ± 83.3 | 0.22 ± 0.01 | 74.7 ± 10.9 | 26.62 ± 5.22 | 16.88 ± 3.75 | 20.6 | 50.9 | 28.5 |
| Cry.tho | 10040.25 ± 2866.8 | 0.08 ± 0.02 | 20 ± 4.6 | 22.81 ± 3.96 | 16.01 ± 2.09 | 56.5 | 0.0 | 43.5 |
| Dig.mic | 3718.4 ± 1205.1 | 0.17 ± 0.04 | 44.8 ± 9.2 | 18 ± 2.77 | 24.45 ± 3.76 | 55.1 | 7.1 | 37.7 |
| Ech.sic | 9063.19 ± 6212.3 | 0.17 ± 0.02 | 142.5 ± 146.4 | 14.84 ± 3.59 | 27.76 ± 6.98 | 42.9 | 40.6 | 16.5 |
| Eup.cor | 1204.9 ± 279.1 | 0.17 ± 0.03 | 19.8 ± 3.1 | 39.75 ± 2.4 | 10.99 ± 1.12 | 27.1 | 6.5 | 66.4 |
| Eup.meu | 1101.1 ± 108.9 | 0.15 ± 0.07 | 93.9 ± 15.3 | 32.01 ± 1.63 | 13.36 ± 0.92 | 34.8 | 3.3 | 61.9 |
| Eup.sem | 236.55 ± 302.6 | 0.29 ± 0.02 | 35.1 ± 7.6 | 21.77 ± 1.27 | 20.09 ± 1.04 | 10.2 | 42.2 | 47.5 |
| Gle.sar | 731.55 ± 11436.7 | 0.15 ± 0.03 | 40.2 ± 17.3 | 18.82 ± 2.03 | 20.96 ± 1.61 | 33.6 | 1.9 | 64.5 |
| Gym.edd | 11850.9 ± 13147.1 | 0.17 ± 0.03 | 36.1 ± 8.4 | 29.4 ± 5.77 | 13.09 ± 0.99 | 64.1 | 4.2 | 31.7 |
| Hel.boc | 9901 ± 10894.9 | 0.18 ± 0.04 | 464.9 ± 454.6 | 34.2 ± 6.30 | 13.36 ± 2.31 | 33.7 | 52.4 | 13.9 |
| Hep.ang | 8993.9 ± 316.1 | 0.16 ± 0.03 | 52.4 ± 13.4 | 16.52 ± 1.23 | 23.04 ± 2.03 | 70.2 | 2.9 | 26.9 |
| Hyp.hir | 1124.6 ± 1059.1 | 0.22 ± 0.03 | 32.6 ± 7.2 | 30.25 ± 1.23 | 15.05 ± 0.73 | 26.4 | 25.3 | 48.4 |
| Kla.cic | 2896.95 ± 1633.1 | 0.18 ± 0.03 | 67.3 ± 7.4 | 16.66 ± 2.77 | 26.19 ± 1.94 | 58.4 | 18.4 | 23.2 |
| Kna.gus | 3683.9 ± 1121.9 | 0.18 ± 0.03 | 44.5 ± 6 | 21.37 ± 4.05 | 21.29 ± 2.75 | 52.6 | 11.4 | 36.0 |
| Kna.luc | 4597.3 ± 2636.2 | 0.19 ± 0.03 | 49.5 ± 9.5 | 13.91 ± 2.71 | 32.09 ± 5.59 | 54.8 | 14.3 | 30.9 |
| Lat.jor | 972.3 ± 288.5 | 0.19 ± 0.02 | 34.1 ± 4.5 | 43.18 ± 1.3 | 10.4 ± 0.42 | 28.9 | 16.2 | 54.9 |
| Luz.sic | 305.05 ± 244.3 | 0.34 ± 0.43 | 31.7 ± 69.6 | 22.32 ± 3.93 | 19.9 ± 3.13 | 12.9 | 28.8 | 58.3 |
| Mal.cre | 959.8 ± 213.9 | 0.25 ± 0.07 | 31.7 ± 8.7 | 25.73 ± 6.73 | 17.36 ± 2.91 | 21.9 | 32.0 | 46.2 |
| Mel.ita | 562.9 ± 170.2 | 0.2 ± 0.02 | 34 ± 4.4 | 20.53 ± 8.75 | 19.94 ± 1.42 | 21.9 | 20.2 | 57.9 |
| Myo.flo | 449.05 ± 190.0 | 0.16 ± 0.05 | 25.8 ± 7.2 | 27.73 ± 11.27 | 14.69 ± 5.65 | 19.1 | 6.9 | 74.1 |
| Pae.san | 20502.3 ± 11295.3 | 0.25 ± 0.04 | 47.8 ± 19.6 | 19.91 ± 1.62 | 19.09 ± 1.6 | 63.3 | 18.5 | 18.2 |
| Pet.gus | 3630.2 ± 2644.0 | 0.1 ± 0.04 | 18.6 ± 4.4 | 24.11 ± 7.24 | 16.71 ± 2.37 | 38.6 | 0.5 | 60.9 |
| Phy.pse | 1532.7 ± 1060.3 | 0.16 ± 0.04 | 27.4 ± 5.3 | 23.69 ± 9.09 | 19.06 ± 2.66 | 32.6 | 7.4 | 59.9 |
| Pin.lar | 226.95 ± 51.6 | 0.52 ± 0.05 | 281.8 ± 49.3 | 8.91 ± 1.51 | 50.35 ± 8.56 | 8.4 | 91.6 | 0 |
| Rha.per | 845.55 ± 20178.5 | 0.3 ± 0.06 | 61.8 ± 14.1 | 27.92 ± 8.44 | 15.98 ± 2.77 | 22.6 | 52.1 | 25.3 |
| Rha.cen | 30248.3 ± 394.1 | 0.2 ± 0.06 | 56.7 ± 29.8 | 24.85 ± 5.85 | 16.11 ± 2.29 | 72.3 | 14.7 | 13.0 |
| Sal.arr | 945.85 ± 460.2 | 0.49 ± 0.15 | 75.4 ± 17.6 | 21.38 ± 1.24 | 22.2 ± 1.77 | 18.0 | 70.7 | 11.3 |
| Sal.gus | 1088.7 ± 261.8 | 0.42 ± 0.02 | 93 ± 14 | 18.48 ± 1.11 | 23.14 ± 2.19 | 22.8 | 72.5 | 4.7 |
| Sym.gus | 1400.4 ± 395.5 | 0.09 ± 0.01 | 21.4 ± 4.5 | 37.3 ± 5.24 | 10.97 ± 1.35 | 31.7 | 0.0 | 68.3 |
| Tep.ita | 3740.6 ± 1165.9 | 0.16 ± 0.04 | 56.8 ± 59.2 | 17.22 ± 3.26 | 23.82 ± 4.54 | 56.0 | 7.2 | 36.8 |
| Vic.bru | 4328.77 ± 1289.0 | 0.27 ± 0.02 | 42.3 ± 7.7 | 32.08 ± 4.42 | 12.58 ± 1.58 | 41.7 | 30.7 | 27.7 |
| Vic.och | 1258.2 ± 220.0 | 0.27 ± 0.02 | 32.4 ± 3.7 | 42.88 ± 4.22 | 10.33 ± 0.69 | 24.5 | 34.6 | 41.0 |
| **Woody** | 1641.9 ± 2493.3 | 0.38 ± 0.24 | 101.7 ± 102.7 | 23.10 ± 9.78 | 25.37 ± 16.67 | 22.6 | 55.6 | 21.8 |
| **Herbs** | 5497.4 ± 9497.5 *** | 0.20 ± 0.16*** | 53.7 ± 113.7*** | 28.03 ± 10.56* | 16.67± 6.20*** | 39.5** | 18.1*** | 42.4** |

| Variable | Intercept | Estimate | P value | R^2^marginal | R^2^conditional |
| --- | --- | --- | --- | --- | --- |
| LA woody | 5127.367 | -894.542 | 0.0198* | **0.003** | **0.8305** |
| LA herb | 6573.99 | -2749.06 | 5.48e-11*** | 0.022 | 0.628 |
| LDMC woody | 0.38573 | -0.02122 | 0.124 | 0.003 | 0.502 |
| LDMC herbs | 2.206e-01 | 2.281e-03 | 0.812 | 5.900606e-05 | 0.221 |
| LMA woody | 6.991e-02 | 1.033e-02 | 0.00809** | 0.006 | 0.701 |
| LMA herb | 3.816e-02 | 2.924e-03 | 0.00568** | 0.005 | 0.492 |
| Nmass woody | 22.2285 | 0.8031 | 0.449 | 0.002 | 0.666 |
| Nmass herbs | 27.7843 | 1.9655 | 0.028* | 0.009 | 0.649 |
| C:N woody | 24.3597 | -0.4667 | 0.54913 | 0.0002 | 0.953 |
| C:N herb | 17.5545 | -1.6736 | 0.00278** | 0.014 | 0.711 |
| C woody | 29.3009 | -1.5016 | 0.01413* | 0.001 | 0.938 |
| C herb | 39.3321 | -3.4571 | 2.62e-09** | 0.009 | 0.804 |
| S woody | 53.2308 | -0.9218 | 0.496459 | 0.0003 | 0.835 |
| S herb | 22.827 | -5.395 | 1.20e-07** | 0.017 | 0.540 |
| R woody | 17.483 | 2.415 | 0.04208* | 0.005 | 0.597 |
| R herb | 37.8490 | 8.8511 | < 2e-16*** | 0.069 | 0.438 |

**Supplementary Table 4**. Model results of endemic vs non-endemic species pair analysis (n = 27 pairs) showing direction (estimate) and significance of effects on LA, LDMC, Nmass and C:N ratio separately for woody and herbaceous species; effects on competitive ability (C), stress-tolerance (S) and Ruderality (R) are also shown.

| **Supplementary Table 5.** Means ± sd and significance of differences for each trait within each of the 27 endemic vs. non-endemic species pair. | | | | | | | | | | | | | |  |
| --- | --- | --- | --- | --- | --- | --- | --- | --- | --- | --- | --- | --- | --- | --- |
| Species | Pair end | | LA |  | | LDMC | LMA | | Nmass | |  | | C:N |  |
| Abi.alb | 1 | no | 42.29 ± 9.48 | | 0.54 ± 0.04 | | | 150.9 ± 14.4 | | 7.95 ± 0.37 | | 60.53 ± 3.16 | | |
| Abi.neb | 1 | yes | 34.7 ± 4.52 ** | | 0.59 ± 0.31 | | | 241.7 ± 92.4*** | | 7.39 ± 1.06 | | 64.3 ± 8.28 | | |
| Ace.pla | 2 | no | 14259.35 ± 4361.93 | | 0.40 ± 0.03 | | | 59.2 ± 11 | | 26.13 ± 4.9 | | 16.93 ± 3.58 | | |
| Ace.lob | 2 | yes | 8539.8 ± 2545.19 *** | | 0.27 ± 0.04 *** | | | 41.8 ± 9.6*** | | 30.36 ± 8.44 | | 15.17 ± 3.38 | | |
| Aeg.pur | 3 | no | 424.7 ± 86.29 | | 0.21 ± 0.01 | | | 37.2 ± 3.2 | | 26.68 ± 3.24 | | 14.93 ± 1.89 | | |
| Aeg.cal | 3 | yes | 233.85 ± 47.7 *** | | 0.26 ± 0.07 ** | | | 51.6 ± 15.5*** | | 24.08 ± 2.89 | | 16.72 ± 1.71 | | |
| Aqu.dum | 4 | no | 23325.06 ± 11390.68 | | 0.27 ± 0.04 | | | 22.5 ± 3.9 | | 23.93 ± 3.25 | | 17.60 ± 2.79 | | |
| Aqu.bar | 4 | yes | 4731.1 ± 1731.02 *** | | 0.18 ± 0.04 *** | | | 26.2 ± 8.2 | | 27.81 ± 8.14 | | 14.95 ± 4.21 | | |
| Aqu.dum | 5 | no | 23325.06 ± 11390.68 | | 0.27 ± 0.04 | | | 22.5 ± 3.9 | | 23.93 ± 3.25 | | 17.60 ± 2.79 | | |
| Aqu.nug | 5 | yes | 3738.3 ± 1526.74 *** | | 0.23 ± 0.09 ** | | | 31.9 ± 13.7* | | 27.43 ± 2.1 | | 13.69 ± 0.85 * | | |
| Aqu.dum | 6 | no | 23325.06 ± 11390.68 | | 0.27 ± 0.04 | | | 22.5 ± 3.9 | | 23.93 ± 3.25 | | 17.60 ± 2.79 | | |
| Aqu.sic | 6 | yes | 5927.7 ± 3238.06 *** | | 0.18 ± 0.04 *** | | | 42.0 ± 13.1*** | | 35.61 ± 5.56 ** | | 12.14 ± 2.06 ** | | |
| Ari.lut | 7 | no | 1526.8 ± 337.78 | | 0.23 ± 0.02 | | |  | | 32.81 ± 4.5 | | 13.15 ± 2.03 | | |
| Ari.sic | 7 | yes | 2908 ± 1417.75 *** | | 0.43 ± 0.21 ** | | | 58.4 ± 28.7** | | 50.32 ± 3.67 ** | | 8.67 ± 0.61 ** | | |
| Ari.lut | 8 | no | 1526.8 ± 337.78 | | 0.23 ± 0.02 | | | 28.9 ± 4 | | 32.81 ± 4.5 | | 13.15 ± 2.03 | | |
| Ari.tyr | 8 | yes | 905.45 ± 506.52 *** | | 0.19 ± 0.03 *** | | | 38.2 ± 14.6** | | 32.74 ± 7.7 | | 12.56 ± 2.6 | | |
| Bet.pen | 9 | no | 1488.95 ± 720.52 | | 0.27 ± 0.03 | | | 43.4 ± 7.3 | | 25.1 ± 2.24 | | 18.68 ± 1.46 | | |
| Bet.aet | 9 | yes | 945.9 ± 190.2 *** | | 0.3 ± 0.03 *** | | | 48.7 ± 4.1** | | 33.09 ± 3.33 ** | | 13.78 ± 1.9 ** | | |
| Bry.dio | 10 | no | 4793.25 ± 1338.32 | | 0.17 ± 0.02 | | | 35.8 ± 7.2 | | 37.09 ± 9.11 | | 10.01 ± 1.74 | | |
| Bry.mar | 10 | yes | 4354.5 ± 2589.52 | | 0.15 ± 0.04 | | | 27.7 ± 8.9** | | 32.66 ± 5.77 | | 11.86 ± 1.65 | | |
| Car.hep | 11 | no | 23237.89 ± 10294.04 | | 0.18 ± 0.09 | | | 30.5 ± 6.3 | | 47.26 ± 3.55 | | 9.17 ± 0.52 | | |
| Car.bat | 11 | yes | 34197.63 ± 14920.5 * | | 0.06 ± 0.02 *** | | | 15.6 ± 11.7*** | | 38.57 ± 5.27 * | | 10.85 ± 1.17 * | | |
| Cro.neg | 12 | no | 366.9 ± 122.65 | | 0.23 ± 0.03 | | | 69.6 ± 11.5 | | 39.51 ± 0.39 | | 10.11 ± 0.15 | | |
| Cro.etr | 12 | yes | 232.05 ± 83.27 *** | | 0.22 ± 0.01 * | | | 74.7 ± 10.9 | | 26.62 ± 5.22 ** | | 16.88 ± 3.75 * | | |
| Dig.lut | 13 | no | 7045.45 ± 3355.96 | | 0.14 ± 0.03 | | | 45.8 ± 5.9 | | 23.92 ± 3.68 | | 17.44 ± 2.55 | | |
| Dig.mic | 13 | yes | 3718.4 ± 1205.09 *** | | 0.17 ± 0.04 * | | | 44.8 ± 9.2 | | 18 ± 2.77 | | 24.45 ± 3.76 * | | |
| Eup.amy | 14 | no | 769.25 ± 232.97 | | 0.26 ± 0.04 | | | 57.8 ± 15 | | 24.2 ± 7.12 | | 19.16 ± 5.03 | | |
| Eup.meu | 14 | yes | 852.35 ± 355.69 | | 0.37 ± 0.29 | | | 93.9 ± 15.3*** | | 32.01 ± 2.45 | | 13.36 ± 0.92 | | |
| Eup.amy | 15 | no | 769.25 ± 232.97 | | 0.26 ± 0.04 | | | 57.8 ± 15 | | 24.2 ± 7.12 | | 19.16 ± 5.03 | | |
| Eup.sem | 15 | yes | 236.55 ± 302.58 *** | | 0.29 ± 0.02 | | | 35.1 ± 7.6*** | | 21.77 ± 1.27 | | 20.09 ± 1.04 | | |
| Eup.amy | 16 | no | 769.25 ± 232.97 | | 0.26 ± 0.04 | | | 57.8 ± 15 | | 24.2 ± 7.12 | | 19.16 ± 5.03 | | |
| Eup.cor | 16 | yes | 1204.9 ± 279.08 | | 0.17 ± 0.03 ** | | | 19.8 ± 3.1*** | | 39.74 ± 2.45 | | 10.99 ± 1.12 | | |
| Gle.hed | 17 | no | 916.2 ± 213.12 | | 0.16 ± 0.03 | | | 25.4 ± 2.6 | | 24.81 ± 4.64 | | 16.97 ± 1.68 | | |
| Gle.sar | 17 | yes | 731.55 ± 11436.66 * | | 0.15 ± 0.03 * | | | 40.2 ± 17.3*** | | 18.82 ± 2.03 ** | | 20.96 ± 1.61 ** | | |
| Kna.dry | 18 | no | 3959.8 ± 161.36 | | 0.20 ± 0.02 | | | 40.1 ± 7.1 | | 13.35 ± 2.87 | | 33.16 ± 4.14 | | |
| Kna.gus | 18 | yes | 3683.9 ± 1121.96 | | 0.18 ± 0.03 | | | 44.5 ± 6** | | 21.37 ± 4.05 ** | | 21.29 ± 2.75 ** | | |
| Kna.dry | 19 | no | 3959.8 ± 161.36 | | 0.20 ± 0.02 | | | 40.1 ± 7.1 | | 13.35 ± 2.87 | | 33.16 ± 4.14 | | |
| Kna.luc | 19 | yes | 4597.3 ± 2636.22 | | 0.19 ± 0.03 | | | 49.5 ± 9.5** | | 13.91 ± 2.71 | | 32.09 ± 5.59 | | |
| Lat.nig | 20 | no | 1448.1 ± 344.94 | | 0.24 ± 0.02 | | | 29.5 ± 2.7 | | 36.21 ± 5.66 | | 13.02 ± 2.26 | | |
| Lat.jor | 20 | yes | 972.3 ± 288.49 *** | | 0.19 ± 0.02 | | | 34.1 ± 4.5*** | | 43.18 ± 1.3 * | | 10.38 ± 0.42 | | |
| Luz.syl | 21 | no | 1139.55 ± 1058.28 | | 0.31 ± 0.04 | | | 47.1 ± 8.9 | | 15.61 ± 2.16 | | 28.73 ± 4.46 | | |
| Luz.sic | 21 | yes | 305.05 ± 244.35 ** | | 0.34 ± 0.43 ** | | | 31.7 ± 69.6*** | | 22.32 ± 3.93 ** | | 19.9 ± 3.13 ** | | |
| Mal.syl | 22 | no | 2098.25 ± 795.19 | | 0.35 ± 0.03 | | | 39.2 ± 3.8 | | 19.31 ± 6.88 | | 23.75 ± 1.45 | | |
| Mal.cre | 22 | yes | 959.8 ± 213.93 *** | | 0.25 ± 0.07 *** | | | 31.7 ± 8.7* | | 25.73 ± 6.73 | | 17.36 ± 2.91 ** | | |
| Pae.mas | 23 | no | 20209.78 ± 4021.27 | | 0.21 ± 0.02 | | | 56 ± 7.1 | | 27.23 ± 3.16 | | 16.21 ± 1.62 | | |
| Pae.san | 23 | yes | 20502.3 ± 11295.33 | | 0.25 ± 0.04 ** | | | 47.8 ± 19.6 | | 19.91 ± 1.62 ** | | 19.09 ± 1.6 * | | |
| Rha.cat | 24 | no | 1024.65 ± 207.32 | | 0.31 ± 0.04 | | | 47.4 ± 10.1 | | 26.78 ± 1.63 | | 15.84 ± 1.05 | | |
| Rha.per | 24 | yes | 845.55 ± 20178.49 ** | | 0.30 ± 0.06 | | | 61.8 ± 14.1 | | 27.92 ± 8.44 | | 16.71 ± 2.77 | | |
| Sal.atr | 25 | no | 764.75 ± 339 | | 0.49 ± 0.19 | | | 85.9 ± 25.4 | | 22.67 ± 2.91 | | 21.47 ± 2.95 | | |
| Sal.arr | 25 | yes | 945.85 ± 460.15 | | 0.49 ± 0.15 | | | 75.4 ± 17.6 | | 21.38 ± 1.24 | | 22.2 ± 1.77 | | |
| Sal.atr | 26 | no | 764.75 ± 339 | | 0.49 ± 0.19 | | | 85.9 ± 25.4 | | 22.67 ± 2.91 | | 21.47 ± 2.95 | | |
| Sal.gus | 26 | yes | 1088.7 ± 261.82 ** | | 0.42 ± 0.02 | | | 93 ± 14 | | 18.48 ± 1.11 * | | 23.14 ± 2.19 | | |
| Sym.tub | 27 | no | 3217.75 ± 1393.64 | | 0.11 ± 0.01 | | | 25.2 ± 3.7 | | 40.1 ± 2.49 | | 10.23 ± 0.77 | | |
| Sym.gus | 27 | yes | 1400.4 ± 395.48 ** | | 0.09 ± 0.01 *** | | | 21.4 ± 4.5** | | 37.3 ± 5.24 | | 10.97 ± 1.35 | | |

| **Supplementary Table 6.** Mean values ± sd of C, S and R strategies and significance of differences within each of the 27 endemic vs. non-endemic species pairs. | | | | | |
| --- | --- | --- | --- | --- | --- |
| Species | pair | Endem | C% | S% | R% |
| Abi.alb | 1 | no | 1.29 ± 0.68 | 98.71 ± 0.68 | 0.00 |
| Abi.neb | 1 | yes | 1.20 ± 0.78 | 98.85 ± 0.78 | 0.00 |
| Ace.pla | 2 | no | 51.08 ± 4.77 | 37.41 ± 2.86 | 11.51 ± 4.1 |
| Ace.lob | 2 | yes | 50.23 ± 5.18 | 25.65 ± 7.85 *** | 24.13 ± 7.57 *** |
| Aeg.pur | 3 | no | 18.67 ± 2.67 | 27.42 ± 5.14 | 53.91 ± 4.82 |
| Aeg.cal | 3 | yes | 13.31 ± 3.25 *** | 45.31 ± 20.7 *** | 41.38 ± 18.41 ** |
| Aqu.dum | 4 | no | 55.68 ± 5.96 | 17.69 ± 5.22 | 26.63 ± 4.16 |
| Aqu.bar | 4 | yes | 46.67 ± 7.47 | 6.35 ± 10.39 *** | 46.98 ± 12.02 *** |
| Aqu.dum | 5 | no | 55.68 ± 5.96 | 17.69 ± 5.22 | 26.63 ± 4.16 |
| Aqu.nug | 5 | yes | 39.33 ± 9.54 *** | 20.52 ± 18.57 | 40.15 ± 15.11 *** |
| Aqu.dum | 6 | no | 55.68 ± 5.96 | 17.69 ± 5.22 | 26.63 ± 4.16 |
| Aqu.sic | 6 | yes | 56.53 ± 12.51 | 8.86 ± 11.3 ** | 34.61 ± 12.56 |
| Ari.lut | 7 | no | 28.26 ± 2.73 | 24.88 ± 5.63 | 46.86 ± 4.51 |
| Ari.sic | 7 | yes | 28.08 ± 7.29 | 43.51 ± 30.53 * | 28.41 ± 27.17 * |
| Ari.lut | 8 | no | 28.26 ± 2.73 | 24.88 ± 5.63 | 46.86 ± 4.51 |
| Ari.tyr | 8 | yes | 27.73 ± 6 | 19.15 ± 14.44 | 53.13 ± 12.94 * |
| Bet.pen | 9 | no | 28.19 ± 5.72 | 38.11 ± 7.53 | 33.7 ± 7.44 |
| Bet.aet | 9 | yes | 22.59 ± 2.82 *** | 48.16 ± 5.59 *** | 29.25 ± 4 |
| Bry.dio | 10 | no | 54.51 ± 5.72 | 4.77 ± 6.13 | 40.72 ± 7.1 |
| Bry.mar | 10 | yes | 46.88 ± 10.09 ** | 3.76 ± 6.54 | 49.35 ± 10.5 ** |
| Car.hep | 11 | no | 68.89 ± 8.33 | 4.52 ± 8.4 | 26.59 ± 6.02 |
| Car.bat | 11 | yes | 63.22 ± 11.19 | 3.34 ± 13.34 | 33.44 ± 11.61 * |
| Cro.neg | 12 | no | 22.51 ± 4.74 | 48.88 ± 13.85 | 28.61 ± 12.27 |
| Cro.etr | 12 | yes | 20.62 ± 4.82 | 50.87 ± 9.7 | 28.5 ± 9.49 |
| Dig.lut | 13 | no | 66.21 ± 7.93 | 1.71 ± 2.87 | 32.08 ± 7.12 |
| Dig.mic | 13 | yes | 55.14 ± 8.92 *** | 7.14 ± 11.38 | 37.72 ± 9.73 * |
| Eup.amy | 14 | no | 25.16 ± 4.21 | 44.81 ± 14.2 | 30.03 ± 13.39 |
| Eup.cor | 14 | yes | 27.14 ± 6.39 | 6.45 ± 8.37 *** | 66.41 ± 8.72 *** |
| Eup.amy | 15 | no | 25.16 ± 4.21 | 44.81 ± 14.2 | 30.03 ± 13.39 |
| Eup.meu1 | 15 | yes | 34.8 ± 4.64 *** | 3.29 ± 14.73 *** | 61.91 ± 12.37 *** |
| Eup.amy | 16 | no | 25.16 ± 4.21 | 44.81 ± 14.2 | 30.03 ± 13.39 |
| Eup.sem | 16 | yes | 10.22 ± 8.63 *** | 42.24 ± 8.52 | 47.54 ± 12.26 *** |
| Gle.hed | 17 | no | 28.21 ± 2.51 | 2.86 ± 6.57 | 68.93 ± 6.06 |
| Gle.sar | 17 | yes | 33.58 ± 7.1 ** | 1.91 ± 6.8 | 64.51 ± 4.4 |
| Kna.dry | 18 | no | 48.46 ± 2.32 | 15.01 ± 5.11 | 36.53 ± 4.5 |
| Kna.gus | 18 | yes | 52.58 ± 6.38 | 11.38 ± 8.16 | 36.03 ± 6.54 |
| Kna.dry | 19 | no | 48.46 ± 2.32 | 15.01 ± 5.11 | 36.53 ± 4.50 |
| Kna.luc | 19 | yes | 54.79 ± 12.09 | 14.34 ± 12.55 | 30.87 ± 9.63 * |
| Lat.nig | 20 | no | 27.04 ± 3.38 | 27.59 ± 4.86 | 45.37 ± 3.35 |
| Lat.jor | 20 | yes | 28.91 ± 3.88 | 16.23 ± 8.37 *** | 54.86 ± 6.97 *** |
| Luz.syl | 21 | no | 21.2 ± 12.59 | 48.51 ± 8.48 | 30.28 ± 7.59 |
| Luz.sic | 21 | yes | 12.91 ± 8.61 * | 28.79 ± 24.41 ** | 58.29 ± 18.32 *** |
| Mal.syl | 22 | no | 25.91 ± 3.44 | 46.1 ± 3.38 | 28 ± 3.91 |
| Mal.cre | 22 | yes | 21.87 ± 2.81 *** | 31.98 ± 15.44 *** | 46.15 ± 14.87 *** |
| Pae.mas | 23 | no | 73.42 ± 2.93 | 11.83 ± 3.43 | 14.75 ± 3.52 |
| Pae.san | 23 | yes | 63.3 ± 9.82 *** | 18.47 ± 8.14 ** | 18.23 ± 8.36 |
| Rha.cat | 24 | no | 22.62 ± 2.28 | 47.85 ± 8.25 | 29.53 ± 7.86 |
| Rha.per | 24 | yes | 22.6 ± 14.56 | 52.1 ± 16.89 | 25.29 ± 4.75 |
| Sal.atr | 25 | no | 17.62 ± 6.76 | 71.42 ± 20.56 | 10.96 ± 15.41 |
| Sal.arr | 25 | yes | 18.02 ± 5.21 | 70.72 ± 9.4 | 11.26 ± 7.62 |
| Sal.atr | 26 | no | 17.62 ± 6.76 | 71.42 ± 20.56 | 10.96 ± 15.41 |
| Sal.gus | 26 | yes | 22.82 ± 2.79 * | 72.46 ± 4.47 | 4.72 ± 4.58 *** |
| Sym.tub | 27 | no | 44.52 ± 7.09 | 0.00 | 55.48 ± 7.09 |
| Sym.gus | 27 | yes | 31.68 ± 4.17 *** | 0.00 | 68.32 ± 4.17 *** |
